# Multi-Strategy Fusion Improved Walrus Optimization Algorithm for Coverage Optimization in Wireless Sensor Networks

**DOI:** 10.3390/biomimetics11010072

**Published:** 2026-01-15

**Authors:** Ling Li, Youyi Ding, Xiancun Zhou, Xuemei Zhu, Zongling Wu, Wei Peng, Jingya Zhang, Chaochuan Jia

**Affiliations:** 1 School of Electronic Information and Artificial Intelligence, West Anhui University, Lu’an 237012, China; 2 School of Electrical and Optoelectronic Engineering, West Anhui University, Lu’an 237012, China; 3 Department of Experimental and Practical Training Teaching Management, West Anhui University, Lu’an 237012, China

**Keywords:** walrus optimization, differential evolution, Logistics–Sine–Cosine Mapping, beta opposition-based learning, wireless sensor networks

## Abstract

The Walrus Optimization (WO) algorithm, a metaheuristic inspired by walrus behavior, is known for its competitive convergence speed and effectiveness in solving high-dimensional and practical engineering optimization problems. However, it suffers from a tendency to converge to local optima and exhibits instability during the iterative process. To overcome these limitations, this study proposes an improved WO (IMWO) algorithm based on the integration of Differential Evolution/best/1 (DE/best/1) mutation, Logistics–Sine–Cosine (LSC) Mapping, and the Beta Opposition-Based Learning (Beta-OBL) strategy. These strategies work synergistically to enhance the algorithm’s global exploration capability, improve its search stability, and accelerate convergence with higher precision. The performance of the IMWO algorithm was comprehensively evaluated using the CEC2017 and CEC2022 benchmark test suites, where it was compared against the original WO algorithm and six other state-of-the-art metaheuristics. Experimental data revealed that the IMWO algorithm achieved average fitness rankings of 1.66 and 1.33 in the two test suites, ranking first among all compared algorithms. The WSN coverage optimization problem aims to maximize the monitored area while reducing perception blind spots under limited node resources and energy constraints, which is a typical complex optimization problem with multiple constraints. In a practical application addressing the coverage optimization problem in Wireless Sensor Networks (WSNs), the IMWO algorithm attained average coverage rates of 95.86% and 96.48% in two sets of coverage experiments, outperforming both the original WO and other compared algorithms. These results confirm the practical utility and robustness of the IMWO algorithm in solving complex real-world engineering problems.

## 1. Introduction

Against the backdrop of the rapidly evolving Internet of Things (IoT) technology, WSNs, as the core carrier of information perception and transmission, have been deeply integrated into many key areas, such as smart agriculture and smart cities [1,2,3]. The coverage quality of WSNs directly impacts the comprehensiveness of data collection and the credibility of decision making. Their core optimization goal is to maximize the coverage of monitoring areas under limited node resources and energy constraints, while reducing perceived blind spots. However, traditional random deployment or static layout strategies often encounter problems such as uneven coverage and rapid energy consumption when facing large-scale networks or complex terrain, making it difficult to fully meet the application requirements of practical scenarios [4]. Therefore, optimizing the deployment strategy of sensor nodes through the application of intelligent optimization algorithms has become a core research topic for enhancing the performance of WSNs.

Metaheuristic algorithms, with their powerful global optimization capabilities, have been widely applied in the field of WSN coverage optimization [5]. Swarm intelligence optimization algorithms represent an important branch of metaheuristic algorithms inspired by the collaborative behaviors of swarm organisms in nature (such as ant colonies, bee colonies, and bird flocks) [6]. Their core characteristic is the achievement of global optimization through local interactions among multiple individuals, with information sharing and collaboration among individuals being key. As research deepens, new algorithms continue to emerge. In classic algorithms, Kennedy and Eberhart proposed the particle swarm optimization (PSO) algorithm [7] inspired by the foraging behavior of birds, but it tends to fall into local optima due to the decline in population diversity in the later stage. The cuckoo search (CS) algorithm proposed by Yang relies on Lévy flight to update the position [8], but the convergence accuracy is significantly affected by the flight step size. The gray wolf optimizer (GWO) [9] algorithm and whale optimization algorithm (WOA) [10] proposed by Mirjalili et al. imitate gray wolf hunting and humpback whale bubble net hunting, respectively, but they have the problem of low search efficiency in the later stage. Dorigo et al.’s ant colony optimization (ACO) algorithm [11] and Karaboga’s artificial bee colony (ABC) algorithm [12] perform well in combinatorial optimization, but exhibit insufficient adaptability when applied to WSN coverage optimization in continuous space. In emerging algorithms, Xiao et al.’s artificial lemming algorithm (ALA) [13], Almutairi and Shaheen’s kangaroo escape optimization technique (KET) [14], Martínez Gámez et al.’s tetragonula carbonaria optimization algorithm (TGCOA) [15], Sánchez Cortez et al.’s mantis shrimp optimization algorithm (MShOA) [16], etc., although imitating unique natural behaviors, focus on improving a single search mechanism and fail to effectively balance the dynamic relationship between exploration and exploitation. Xiao et al. proposed the multi-strategy boosted snow ablation optimizer (MSAO) [17] and the joint opposite selection-based arithmetic artificial rabbits optimization algorithm (MAROAOA) [18], which imitate unique natural behaviors and integrate multiple strategies. However, they still have limitations: MSAO tends to suffer from insufficient local exploitation precision in the late iteration stage, while MAROAOA incurs excessive computational overhead due to its complex opposite selection mechanism, resulting in difficulty in effectively balancing the dynamic trade-off between exploration and exploitation. Among them, the WO algorithm [19] proposed by Han et al. searches for the optimal solution through two stages of migration (exploration) and reproduction (exploitation). The structure is simple and easy to implement, but it has significant flaws: First, population update relies on the historical optimal information of individuals, which can easily lead to a lack of population diversity in the later stages of iteration, and it is difficult to jump out of the local optimum in multi-peak coverage scenarios. Second, the risk factors adopt a linear decreasing mode with a fixed variation pattern, resulting in insufficient exploration in the early stage and a tendency to stagnate in the later stage.

In recent years, many swarm intelligence optimization algorithms have been applied to research on coverage optimization in WSNs. For example, Liao et al. used the glowworm swarm optimization (GSO) algorithm [20] to expand the post-deployment coverage area, but failed to address the premature convergence problem. Saravanan et al. introduced the WO algorithm into WSN node coverage enhancement technology [21], but did not mitigate the inherent defects of the WO algorithm itself. Das et al. proposed the termite colony optimization (TCO) algorithm [22] to balance coverage and the number of sensors. Deif et al. combined the local search ACO algorithm [23] to optimize deployment cost and reliability. Mohar et al. improved coverage based on the bat algorithm (BA) [24]. However, these studies were mostly based on the direct application of basic algorithms and lacked ideas for systematic improvement. To overcome the bottleneck of traditional algorithms, scholars have proposed a variety of multi-strategy improvement schemes, but there are still obvious shortcomings: Chen et al. proposed a multi-strategy improved sparrow search algorithm (IM-DTSSA) [25] for WSN coverage optimization, but the strategy fusion lacks synergy and the global exploration breadth is insufficient. Li et al. proposed the virtual force-guided improved sand cat swarm optimization (VF-ISCSO) [26]; although it can reduce coverage blind spots, it relies too heavily on the virtual force mechanism and has limited local development accuracy. Chang et al. proposed a variant of the tuna swarm optimization algorithm based on the behavior evaluation and simplex strategy (SITSO) [27]; it has slow convergence and poor adaptability to multimodal scenarios. Wang et al. introduced a novel self-adaptive multi-strategy artificial bee colony (SaMABC) [28] that enhances optimization performance but has insufficient population diversity maintenance capabilities and is prone to late convergence stagnation. Liang et al. designed a new adaptive Cauchy variant butterfly optimization algorithm (ACBOA) [29], which can improve network coverage, but the dynamic balance control of exploration and development is insufficient. Deepa et al. proposed the Lévy Flight mechanism and the whale optimization algorithm (LWOA) [30], which enhance global exploration capabilities but ignore the collaborative improvement of local fine search and convergence stability.

In view of the shortcomings of the WO algorithm and those of existing improved algorithms, this study proposes an IMWO algorithm that integrates multiple enhancement strategies and applies it to WSN coverage optimization. The core improvements include three collaborative strategies: LSC Mapping [31] is adopted to increase the randomness through the ergodicity and pseudo-randomness of chaotic sequences, which introduces disturbance to expand population diversity. This helps maintain the global search intensity in the early stages while preserving local development disturbance in later stages, thereby balancing exploration and exploitation and improving the algorithm’s convergence accuracy and stability. The DE/best/1 mutation strategy [32] is introduced, drawing on the mutation mechanism based on the optimal individual in the differential evolution algorithm to enhance the algorithm’s ability to escape local optima and improve global search performance. It integrates Beta-OBL [33], which generates opposite solutions of current solutions to provide high-quality candidate individuals, thereby accelerating the convergence speed and improving coverage optimization efficiency.

Compared with existing multi-strategy algorithms, the distinctiveness of IMWO lies not in the simple superposition of strategies, but in the collaborative interaction of its three mechanisms to address core algorithmic deficiencies. Specifically, the DE/best/1 mutation compensates for the insufficient global exploration in algorithms such as IM-DTSSA and SaMABC. The LSC Mapping overcomes the weak maintenance of population diversity in algorithms such as SaMABC. The Beta-OBL strategy mitigates the slow convergence and poor stability observed in algorithms such as ISCSO and LWOA, ultimately achieving a dynamic balance between exploration and development capabilities. Research has found that not only does the IMWO algorithm show better overall performance in function optimization, but its joint improvement strategy can also effectively improve global search capabilities and convergence efficiency. In WSN coverage optimization, the coverage rate achieved by IMWO is significantly higher than that of the comparison algorithms, validating its superiority in solving practical problems. This study provides an efficient solution for improving the perceived performance of WSNs by improving the WO algorithm and applying it to WSN coverage optimization.

The main contributions of this study are summarized as follows:

(1) The proposal of an IMWO algorithm by integrating DE/best/1 mutation, LSC Mapping, and Beta-OBL strategies, which effectively solves the defects of the original WO algorithm, such as easily falling into local optima and insufficient stability.
(2) Establishing a complete mathematical model for WSN coverage optimization, including an objective function, constraint equations, and an encoding scheme, provides a theoretical basis for the application of intelligent optimization algorithms in this field.
(3) Conducting comprehensive experiments on CEC2017/2022 benchmark suites and WSN coverage scenarios, verifying the superiority of IMWO in terms of scalability, robustness, and practical application value.

The remainder of this paper is organized as follows: Section 2 introduces the basic principles of WO. Section 3 presents the basic principles of IMWO. Section 4 validates the effectiveness of IMWO using the CEC2017 and CEC2022 standard function test suites. Section 5 introduces the wireless sensor network coverage model and provides experimental analysis and discussion on the application of IMWO to the WSN coverage problem. Section 6 concludes the paper and outlines future work.

## 2. Walrus Optimization Algorithm, WO

In 2024, M. Han et al. [19], inspired by the natural behaviors of walrus populations, proposed the WO algorithm. Specifically, upon receiving key signals such as danger and safety signals, walruses make behavioral choices such as migration, reproduction, habitat selection, foraging, aggregation, and escape. The WO algorithm draws ideas from these behaviors. In the mathematical model of this algorithm, the search space refers to the range within which the algorithm explores for optimal solutions, typically represented as a multi-dimensional space defined by the decision variables of the problem. The solution space encompasses all potential solutions, with each solution analogous to the position of a walrus in the search space, representing a potential solution to the problem. It can be a vector, where each component corresponds to the value of a decision variable, although the decision variables are determined by the specific problem. The core task of the WO algorithm is to find the optimal solution in the search space that minimizes or maximizes the objective function of the optimization problem.

### 2.1. Danger Signal and Safety Signal

The walrus swarm dynamically adjusts its behavior through an alert mechanism. The danger signal (*DS*) in WO is defined as shown in formula (1).

(1) DS=O∗K

(2) O=2×(1−t/T)

(3) $$K=2\times \alpha_{1}-1$$

The safety signal (*SS*) is defined as shown in formula (4).

(4) $$SS=\alpha_{2}$$

Here, *O* and *K* are danger factors, *t* denotes the current iteration number, and *T* denotes the maximum number of iterations allowed in the process. Additionally, both parameters $\alpha_{1}$ and $\alpha_{2}$ are randomly generated values, confined to the interval (0,1).

### 2.2. Migration (Exploration)

When the danger signal value exceeds 1, the walrus herd will migrate to areas more conducive to the survival of their population. During this migration phase of the walrus herd, the update method for their position is shown in formula (5), which specifies the specific rules for adjusting the position of the walrus at this stage.


(5) $$W_{i,j}^{t+1}=W_{i,j}^{t}+MS$$

Among them, $W_{i,j}^{t+1}$ and $W_{i,j}^{t}$ denote the new position and current position of the *i-th* walrus in the *j-th* dimension, respectively.

*MS* represents the movement step length of walruses, which determines the magnitude of positional change. In specific calculations, it is obtained by multiplying the positional difference between two randomly selected guardian walruses by the control factor and the square of a random number within the interval (0,1).

### 2.3. Reproduction (Exploitation)

When studying the reproduction behavior of walrus pods, we mainly consider the two behavior patterns of walruses: roosting on land and foraging underwater.

(1) Roosting Behavior

When the danger signal is less than 1 and the safety signal is greater than 0.5, walrus groups inhabit land. The walrus population is divided into males, females, and juvenile individuals, and reproductive efficiency is enhanced via differentiated strategies.

Male walruses employ the Halton sequence from the quasi-Monte Carlo method to update their positions. The position update of female walruses is influenced by both male walruses and the leading walrus. Juvenile walruses, being at the edge of the population, are vulnerable to predators and update their positions through Levy Flight to evade dangers.

(2) Foraging behavior

When the danger signal is less than 1 and the safety signal is less than 0.5, the walrus herd forages underwater, including fleeing and gathering behaviors. The fleeing behavior is manifested in that walruses flee the current area according to the danger signals transmitted by their companions when the danger signal is greater than 0.5. The gathering behavior is manifested in that walruses collaboratively locate food-enriched areas when the danger signal is less than 0.5.

**Figure 1:**
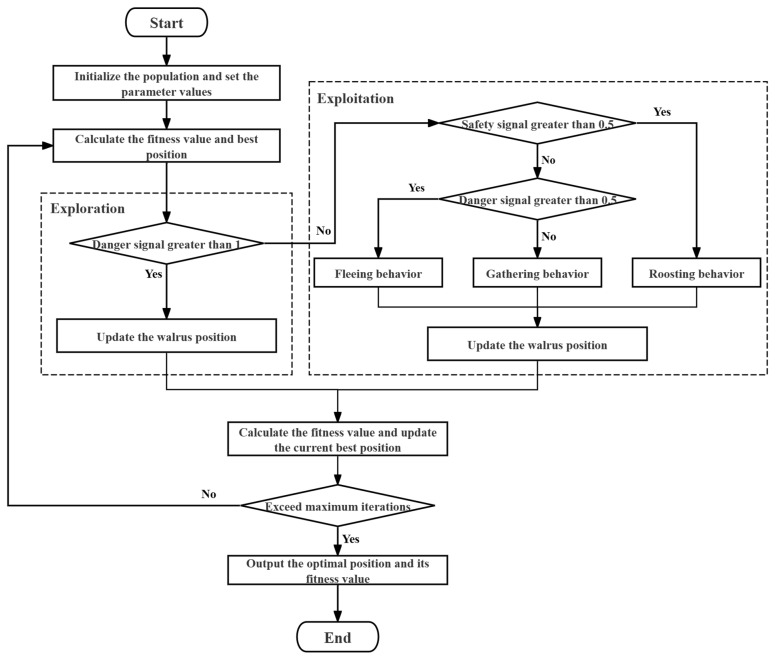
Flow chart of WO.

The flow chart of WO is shown in Figure 1.

## 3. Improved Walrus Optimization Algorithm, IMWO

The WO algorithm, as a metaheuristic algorithm inspired by oceanic phenomena, exhibits advantages such as strong stability and excellent performance in handling high-dimensional and practical problems. However, it suffers from drawbacks such as susceptibility to local optima and insufficient population diversity. To address these issues, this study proposes the IMWO algorithm, which integrates the DE/best/1 mutation, LSC Mapping, and Beta-OBL to achieve multi-strategy collaborative optimization. The incorporation of multiple strategies enables the algorithm to explore the search space from multiple dimensions, effectively compensating for the shortcomings of the WO algorithm. Compared to single-strategy approaches, IMWO enhances global search capability, improves convergence accuracy, and accelerates the convergence through interaction and switching between strategies.

### 3.1. LSC Mapping

LSC Mapping is a hybrid chaotic map that enhances the complexity and randomness of chaotic behavior by combining the nonlinear characteristics of the Logistic map, Sine map, and Cosine map [31]. The recursive formula for the LSC Mapping is shown in (6).

(6) $$m_{t+1}=\cos\left(\pi \cdot \left[4r\cdot m_{t}(1-m_{t})+(1-r)\sin(\pi m_{t})-0.5\right]\right)$$

Here, *r* is a random number uniformly distributed within the interval [0,1], and $m_{t}$ represents the value at the *t-th* iteration. During each iteration, the LSC Mapping dynamically adjusts the contributions of the Logistic map component $4r\cdot m_{t}(1-m_{t})$ and the sine function component $\left(1-r)\sin(\pi m_{t}\right)$ in the overall map based on the value of *r*.

Utilizing the randomness and ergodicity of chaotic sequences, individuals are able to explore the search space more extensively, thereby enhancing the algorithm’s global search capability and increasing the likelihood of escaping local optima. In the traditional WO algorithm, the danger factor *O* is adjusted linearly, which often results in convergence to local optima during later search stages. To address this issue, this study introduces a chaotic sequence to dynamically adjust the danger factor *O*.

In each iteration *t*, *O* is adaptively regulated by the chaotic sequence. The dynamic adjustment formula for parameter *O* is shown in (7).

(7) $$O=\left(2-t\cdot \frac{2}{T}\right)\cdot m_{t+1}$$

where $2-t\cdot \frac{2}{T}$ is a coefficient that linearly decreases as the iteration number *t* increases. The term $m_{t+1}$ introduces chaotic characteristics, endowing parameter *O* with randomness and ergodicity in its values, further enriching the search behavior of the algorithm. During the early optimization stage, when the number of iterations *t* is small and the value of *O* is relatively large, combined with the chaotic randomness of LSC, the position update step size of the walruses exhibits greater diversity, enabling exploration across the entire search space. In the later stage of iteration, *O* gradually decreases as *t* increases. However, LSC Mapping prevents the walrus population from converging entirely to a single region through subtle chaotic fluctuations, maintaining local traversability.

### 3.2. DE/best/1

DE is a population-based stochastic optimization algorithm proposed by Storn and Price in 1995, designed primarily for solving global optimization problems in continuous spaces. The core idea of DE is to generate new candidate solutions through differential information between individuals and then retain better individuals through selection operations, iterating until termination conditions are satisfied. Its basic procedure consists of four steps: Initialization, Mutation, Crossover, and Selection [32].

In the DE algorithm, DE/best/1 is a classic and commonly used mutation strategy, characterized by utilizing the best individual in the current population to guide the mutation direction, thereby enhancing the algorithm’s local search capability and convergence speed. In the reproduction (exploitation) phase, after each position update of the walruses, the DE/best/1 mutation is integrated into the IMWO algorithm. Taking the position of the current population’s best walrus as a benchmark, local perturbation is provided by combining the positional differences in two randomly selected individuals, enabling mutated individuals to generate small fluctuations near the optimal solution and achieve fine-grained search. The DE/best/1 mutation operator formula is shown in (8).

(8) $$W_{i,j}^{t+1}=W_{best}^{t}+G\cdot (W_{r1,j}^{t}-W_{r2,j}^{t})$$

where $W_{best}^{t}$ represents the best individual in the population at the *t-th* iteration. *G* is the scaling factor that controls the magnitude of the difference vector, and it is a pseudo-random number generated within (0, 1) by the rand function. $W_{r1,j}^{t}$ and $W_{r2,j}^{t}$ are two randomly selected different individuals (r1≠r2≠i).

### 3.3. Beta-OBL

Opposition-Based Learning (OBL), proposed by Tizhoosh in 2005 [33], enhances search efficiency by simultaneously evaluating the original solution and its opposite solution. However, traditional OBL employs a deterministic symmetric opposition strategy, which lacks flexibility in complex spaces. Beta-OBL breaks through the deterministic limitations of traditional OBL by incorporating the probabilistic characteristics of the Beta distribution, achieving adaptive exploration of the search space. Its core idea is to dynamically adjust the distribution pattern of opposite solutions based on population diversity, controlling the generation probability of opposite solutions through the shape parameters (*α*, *β*) of the Beta distribution, and balancing the capabilities of global exploration and local exploitation. This study proposes an IMWO algorithm that incorporates a Beta-OBL strategy. In the migration (exploration) phase, the Beta-OBL strategy is introduced after updating the position of the walruses. In the reproduction (exploitation) phase, the Beta-OBL strategy is introduced after applying the DE/best/1 mutation. By generating probabilistic opposition points, it balances global exploration and local exploitation, enhancing the algorithm’s optimization performance in complex spaces.

(1) OBL

Traditional OBL assumes a uniformly distributed search space, and for any solution $W_{i,j}^{t}\in [lb_{j},ub_{j}]$, the constructed inverse solution formula is shown in (9).

(9) $${\overset{⌣}{W}}_{i,j}^{t}=lb_{j}+ub_{j}-W_{i,j}^{t}$$

(2) Beta-OBL

Beta-OBL replaces the uniform distribution with a Beta distribution, controlling the opposite solution’s distribution pattern via shape parameters α and β, allowing the inverse solution to better fit the real search space of the problem. The constructed inverse solution formula is shown in (10).

(10) $${\overset{⌣}{W}}_{i,j}^{t}=(d_{\max,j}-d_{\min,j})\cdot Beta(\alpha ,\beta )+d_{\min,j}$$

(11) $$\alpha =\left\{\begin{array}{c} spread\cdot peak,\\ spread, \end{array}\right.\begin{array}{c} md<0.5\\ otherwise \end{array}$$

(12) $$\beta =\left\{\begin{array}{c} spread,\\ spread\cdot peak, \end{array}\right.\begin{array}{c} md<0.5\\ otherwise \end{array}$$

(13) $$spread=\left\{\begin{array}{c} {\left(\frac{1}{\sqrt{normDiv}}\right)}^{1+N(0,0.5)},\;r_{3}<0.5\\ 0.1\cdot \sqrt{normDiv}+0.9,otherwise \end{array}\right.$$

(14) $$peak=\left\{\begin{array}{c} \frac{(spread-2)\cdot md+1}{spread\cdot (1-md)},\\ \frac{2-spread}{spread}+\frac{spread-1}{spread\cdot md}, \end{array}\right.\begin{array}{c} md<0.5\\ otherwise \end{array}$$

(15) $$md=\left(\begin{array}{c} \frac{d_{\max,j}-W_{i,j}^{t}}{d_{\max,j}-d_{\min,j}},\;r_{3}<0.5\\ \frac{W_{i,j}^{t}-d_{\min,j}}{d_{\max,j}-d_{\min,j}},\;otherwise \end{array}\right.$$

(16) $$normDiv=\frac{1}{N}\sum_{i=1}^{N}\sum_{j=1}^{D}\frac{1}{D}{\left(\frac{W_{i,j}^{t}-{\bar{W}}_{j}}{d_{\max,j}-d_{\min,j}}\right)}^{2}$$

It calculates the dynamic boundaries of the population instead of the fixed boundaries used in the original algorithm, where $d_{\max,j}$ and $d_{\min,j}$ represent the current upper and lower bounds of exploration, respectively. Beta(α,β) is a random variable that follows a Beta distribution with parameters α and β, and it outputs values within the [0,1] interval according to this distribution pattern. $r_{3}$ is a random number uniformly distributed over the interval (0,1). *N* denotes the population size, *D* represents the number of decision variables, and ${\bar{W}}_{j}$ stands for the mean value of all individuals in the *j-th* dimension. After generating reverse solutions, Beta-OBL participates in fitness evaluation alongside candidate solutions from the original IMWO population, and only solutions with better fitness are retained for the next iteration.

**Figure 2:**
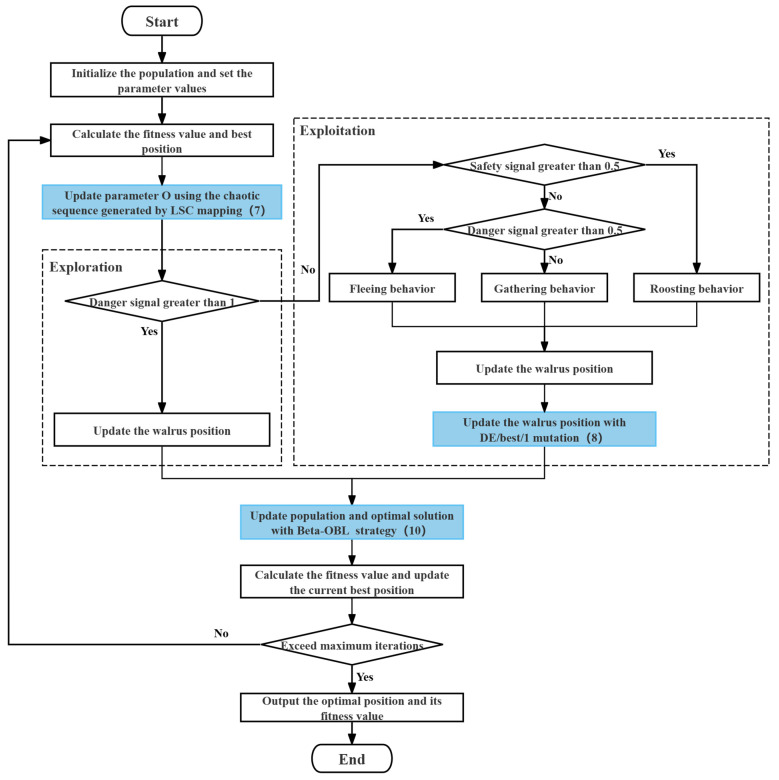
Flow chart of IMWO.

The flow chart of IMWO is shown in Figure 2.

### 3.4. Time Complexity Analysis

Time complexity is the core indicator for evaluating the efficiency of optimization algorithms. The IMWO algorithm adds three new strategies—the LSC Mapping, DE/best/1 mutation, and Beta-OBL strategies—to the WO algorithm framework.

The time complexity of IMWO is still determined by the three core processes of initialization, fitness evaluation, and solution update. Parameter *N* is the population size, *T* is the maximum number of iterations, and *D* is the problem dimension. In the initialization phase, the IMWO algorithm follows the population initialization logic of the WO algorithm, and the complexity remains *O* (*N* × *D*). In the fitness evaluation stage, the IMWO algorithm needs to calculate the fitness values for N search agents one by one in T iterations, with a complexity of *O* (*N* × *T*). None of the three major improvement strategies will change the core process, so there is no essential change in the complexity. The solution update stage is the core difference between the IMWO algorithm and the WO algorithm, but it does not increase the complexity metric level. For the IMWO algorithm’s new chaotic sequence parameter calculations (each iteration *O* (1)), DE/best/1 mutation (traversing the population *O* (*N* × *D*)), and Beta-OBL (population position adjustment *O* (*N* × *D*)), after superimposing T iterations, the total complexity is still determined by the dominant term *O* (*N* × *T* × *D*).

In summary, the IMWO algorithm improves optimization performance while maintaining the time complexity of the WO algorithm, ensuring that the algorithm has good operating efficiency and efficient optimization.

## 4. Experimental Results and Analysis

### 4.1. Experimental Environment

The simulation platform is configured with a Windows 11 operating system, an Intel Core Ultra 7 series processor with a clock speed of 3.80 GHz, and 32 GB of on-board RAM. All algorithms are implemented in MATLAB R2024a.

### 4.2. CEC2017 Test Functions

This section adopts the CEC2017 function set as the test suite. Notably, this suite does not include the F2 function, as it has been explicitly removed by the official due to stability flaws, resulting in a total of 29 test functions. Among them, F1 and F3 are unimodal functions with a single global optimum, which can effectively evaluate the convergence capability of the algorithm. F4–F10 are classified as simple multimodal functions, characterized by the presence of multiple local optima and a single global optimum. These functions are used to test two key capabilities of algorithms, the ability to avoid local optimum traps and the ability to find the global optimum. F11–F20 are hybrid functions, constructed by combining three or more CEC2017 benchmark functions after applying rotations and translations, with each subfunction assigned a certain weight. These functions are primarily used to test the performance of algorithms in handling complex hybrid structural problems. F21–F30 are composition functions, formed by combining at least three hybrid functions or CEC2017 benchmark functions after rotations and translations. Each subfunction has not only a weight but also an additional bias value, which further increases the optimization difficulty for the algorithm.

This study provides a detailed comparison of the performance of eight algorithms on the CEC2017 test functions, including IMWO, WO, the Greylag Goose Optimization (GGO) algorithm [34], the Chinese Pangolin Optimizer (CPO) algorithm [35], the Pigeon-inspired Optimization (PIO) algorithm [36], the Dung Beetle Optimizer (DBO) algorithm [37], the Pelican Optimization algorithm (POA) [38], and the Aquila Optimizer (AO) algorithm [39]. The population size of the test function is set to 30, with a dimensionality of 10, and the number of iterations is 600. The experiment is independently executed 30 times, and the average fitness values of each algorithm are subsequently ranked. Table 1 lists the results for unimodal functions and simple multimodal functions, Table 2 lists the results for hybrid functions, and Table 3 lists the results for composition functions. Among them, min, std, avg, median, and worse represent the optimal value, standard deviation, average value, median, and worst value, respectively.

As shown by the data in Table 1, the average value of IMWO is significantly lower than that of the other seven comparison algorithms on eight functions, including F1, F3–F8, and F10. It has the smallest standard deviation among F1, F3, F4, and F6, with standard deviations on the remaining functions also maintained at a low level. Moreover, the average values and standard deviations of these eight functions are all better than those of the WO algorithm. The DE/best/1 mutation strategy addresses the defect that the WO algorithm is prone to falling into local optima. Through a globally guided mutation step size based on the optimal individual, it enables the IMWO algorithm to jump out of local extreme value regions on functions such as F1 and F5, achieving more accurate global search. The LSC Mapping chaotic perturbation makes up for the deficiency of population convergence in the later stage of the WO algorithm. Leveraging the ergodicity and pseudo-randomness of chaotic sequences, it continuously injects diversity into the population, maintaining exploration vitality in the late iteration stage on functions such as F3 and F7. This ensures that the standard deviation of IMWO remains consistently low, and its stability is far superior to that of the WO algorithm.

As shown by the data in Table 2, in terms of solution accuracy, the IMWO algorithm has significantly lower average values than the other comparison algorithms on functions F16, F17, and F20, while maintaining relatively low average values on the remaining functions. In terms of stability, it achieves the smallest standard deviations on functions F11, F16, and F17, with relatively low standard deviations on other functions. Across all functions F11–F20, the IMWO algorithm exhibits comprehensively lower average values and standard deviations than the original WO algorithm, which mitigates the issues of unstable convergence and insufficient solution accuracy that the WO algorithm is prone to encounter in hybrid functions. The Beta-OBL strategy generates high-quality opposition solutions for the current solutions, constructs a two-way search mechanism, and accelerates the convergence process. This enables the IMWO algorithm to approach the global optimum faster on functions such as F11 and F20, with the convergence speed and solution accuracy far exceeding those of the WO algorithm. The DE/best/1 mutation strategy further enhances the global exploration capability, avoiding the performance bottleneck of the WO algorithm caused by falling into local optima in the complex search space of hybrid functions, and ensuring that the IMWO algorithm maintains stable and excellent performance across the entire set of functions.

As shown by the data in Table 3, the IMWO algorithm achieves significantly lower average values than the other comparison algorithms on five functions, including F21, F23, and F27–F29, and maintains relatively low average values on the remaining functions. In terms of stability, it achieves the smallest standard deviations on functions F21, F22, F27, F29, and F30, with relatively low standard deviations on the other functions. Compared with the WO algorithm, the IMWO algorithm has lower average values on seven functions, including F21, F23, F24, and F27–F30, and smaller standard deviations on six functions, including F21, F22, F26, F27, F29, and F30. Even for the few functions where the WO algorithm has a slight advantage, the overall performance of the IMWO algorithm is more balanced. The chaotic perturbation of LSC Mapping effectively alleviates the problem of the excessively rapid decay of population diversity in the WO algorithm when addressing composition functions. It maintains population vitality during the iteration of F21 and F30, thereby ensuring stability. The DE/best/1 mutation strategy and Beta-OBL strategy operate synergistically: the mutation guided by the optimal individual improves the global search accuracy, while the opposition solutions accelerate convergence. This solves the defects of the WO algorithm in composite functions, such as the imbalance between exploration and exploitation and insufficient convergence accuracy, and achieves the dual optimization of accuracy and stability.

**Figure 3:**
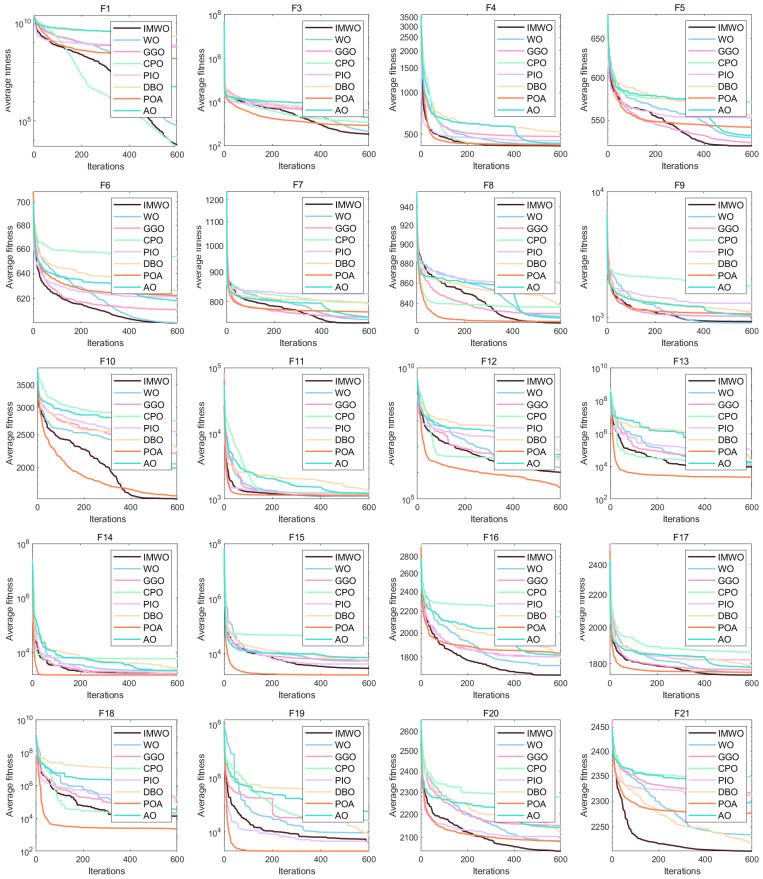

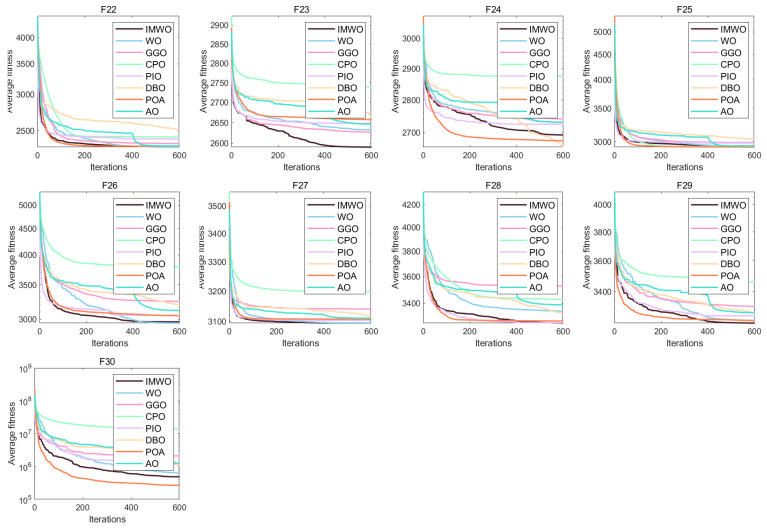
Figure of convergence curve comparison of various algorithms on CEC2017 functions.

Figure 3 displays the convergence curves of all functions from F1 to F30. As observed from the figure, the improved algorithm IMWO outperforms the WO algorithm on most functions, with a faster average fitness decrease and a lower final value, indicating significant improvements in convergence speed and accuracy for IMWO. For example, on functions such as F1 and F5, the IMWO curve lies below that of WO. Compared with other algorithms, IMWO also demonstrates strong competitiveness, leading or ranking among the top performers in most function tests. On functions like F16 and F21, the downward trend of the IMWO fitness curve is significantly better than that of other algorithms. This suggests that the improvement strategies of IMWO are effective and enable better searching for optimal solutions.

**Figure 4:**
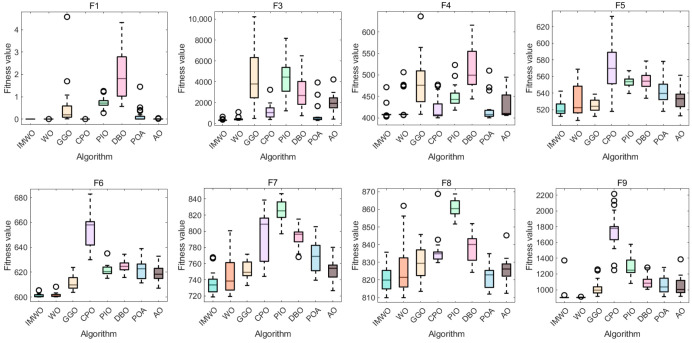

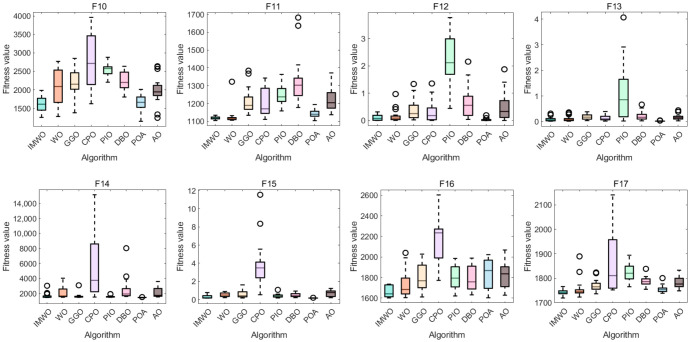

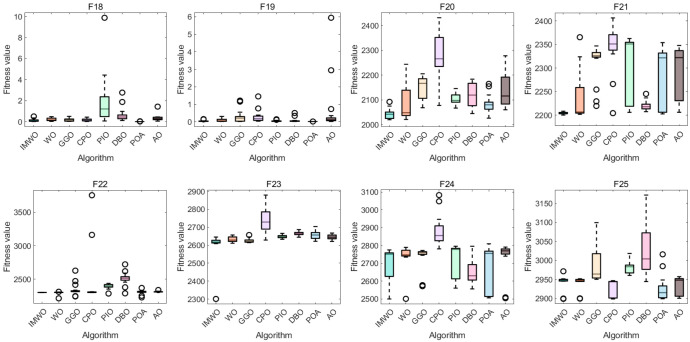

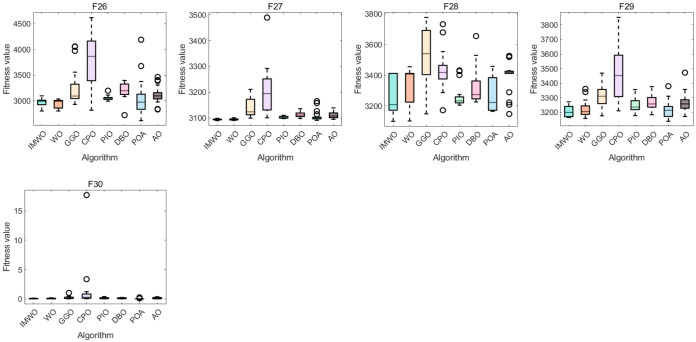
Figure of boxplot comparison of various algorithms on CEC2017 functions.

Figure 4 presents the distribution of fitness values for various algorithms, including WO and IMWO, across the test functions F1 to F30. As evident from the box plots, IMWO outperforms WO in most functions. The boxes corresponding to IMWO are, in most cases, shorter and positioned lower, indicating a more concentrated data distribution and better algorithm stability, which enables it to consistently obtain better solutions across different runs. Other comparative algorithms have their own strengths and weaknesses across different test functions; some outperform IMWO in specific scenarios (e.g., test functions F12 and F13), such as the POA. But overall, IMWO surpasses these algorithms in most cases. This suggests that the improvement strategies effectively enhance the algorithm’s performance, rendering IMWO more advantageous in solving various optimization problems and more stable in finding high-quality solutions.

**Figure 5:**
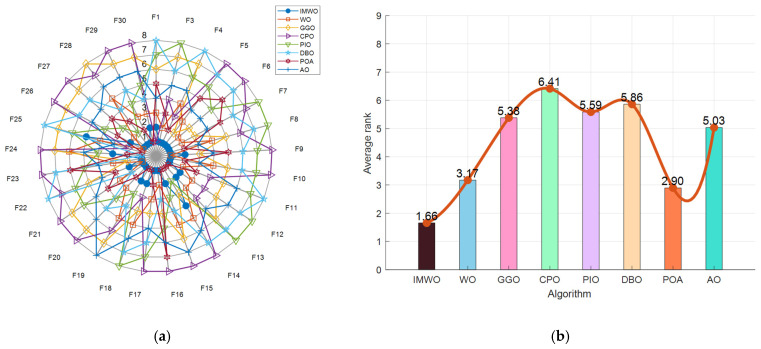
(**a**) Radar chart of various algorithms on CEC2017 functions; (**b**) ranking chart of various algorithms on CEC2017 functions.

Figure 5 presents a performance comparison of WO, IMWO, and other representative algorithms on the CEC2017 benchmark functions, including a radar chart and a ranking bar chart. As can be observed from the radar chart (a), the IMWO algorithm exhibits outstanding performance across multiple functions, with its coverage area surpassing that of WO and other algorithms in numerous function dimensions. This indicates that IMWO demonstrates more balanced and superior performance across different types of functions. The ranking bar chart (b) further quantifies the strengths and weaknesses of each algorithm. With an average ranking of 1.66, IMWO ranks highly among all algorithms, significantly outperforming the original WO algorithm (ranked 3.17). This suggests that the improved IMWO algorithm has significantly enhanced overall performance. Compared to other algorithms, IMWO also showcases strong competitiveness, albeit being slightly inferior to certain algorithms in some function performances. Overall, through the implementation of improved strategies, the problem-solving capabilities of the IMWO algorithm on benchmark functions have been effectively enhanced, demonstrating excellent performance in terms of convergence accuracy, stability, and other aspects. For high-dimensional problems with 30 and 50 dimensions, the comprehensive performance of the IMWO algorithm is also superior to that of the WO algorithm and other comparative algorithms. Detailed statistical data are presented in Figures S1–S4 of the Supplementary Material.

### 4.3. CEC2022 Test Functions

This section adopts the CEC2022 function set as the test suite. The CEC2022 test suite comprises 12 single-objective test functions with boundary constraints, where F1 is a unimodal function, F2–F5 are multimodal functions, F6–F8 are hybrid functions, and F9–F12 are composite and multimodal functions. The population size of the test function is set to 30, with a dimensionality of 10, and the number of iterations is 600. The experiment is run 30 times, and the average fitness values are collected for subsequent analysis. Table 4 presents the results of eight algorithms on the CEC2022 test functions.

According to the data in Table 4, the IMWO algorithm demonstrates excellent performance in both solution accuracy and stability. In terms of solution accuracy, IMWO has significantly lower average values than the other seven comparison algorithms in nine functions, namely F1–F3, F6, F7, and F9–F12. In terms of stability, IMWO performs equally well. It has the smallest standard deviation in nine functions, namely F1–F3, F6–F10, and F12. Most importantly, compared to the WO algorithm, IMWO has comprehensively lower average values and standard deviations in 11 functions, namely F1–F4 and F6–F12. This demonstrates significant improvements over the original algorithm and achieves dual optimization of accuracy enhancement and stability improvement across most test functions.

By introducing the DE/best/1 mutation strategy, IMWO incorporates the perturbation mechanism based on the best individual from differential evolution, which effectively mitigates the WO algorithm’s tendency to fall into local optima. Leveraging globally guided mutation step sizes, it successfully escapes local extrema in multimodal functions F1 and F5, significantly improving the quality of both the average and worst solutions. The LSC chaotic mapping utilizes the ergodicity of chaotic sequences to continuously enhance population diversity, sustaining exploratory vigor in the later stages of functions such as F3 and F7, thereby maintaining a low standard deviation and enhancing algorithmic stability. Beta opposition-based learning generates high-quality opposite candidate solutions, enabling bidirectional search in functions like F2 and F8, further accelerating convergence speed. Consequently, IMWO demonstrates significantly superior minimum and average values across multiple functions compared to competing algorithms.

**Figure 6:**
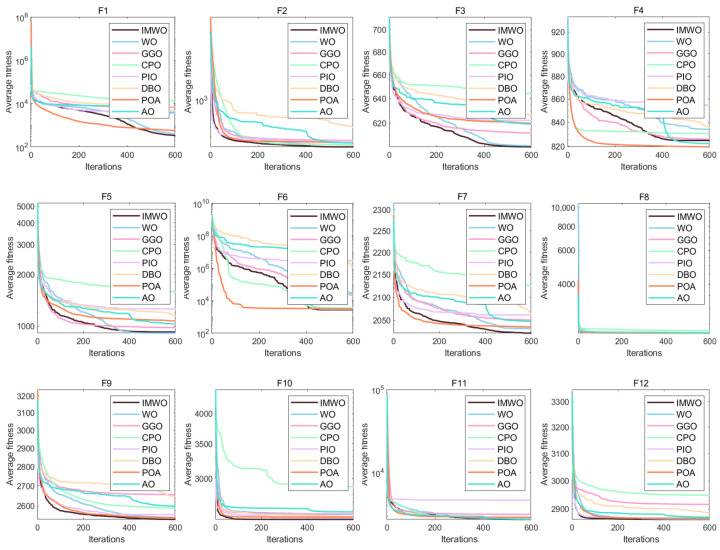
Convergence curve comparison of various algorithms on CEC2022 functions.

Figure 6 displays the variation in the average fitness of eight algorithms across different test functions F1 to F12 with the number of iterations. As can be clearly observed from the figure, IMWO demonstrates superior performance compared to WO and other algorithms in most test functions. Its average fitness value decreases rapidly in the early iterations and stabilizes at a lower level in the later stages, indicating that IMWO has advantages in terms of convergence speed and optimization accuracy. Other algorithms exhibit varying performance across different functions, but overall, the advantage of IMWO is more prominent. This suggests that the improvement strategy effectively enhances the algorithm’s optimization capability, enabling IMWO to quickly converge toward the optimal solution and rendering it more competitive in addressing complex optimization problems.

**Figure 7:**
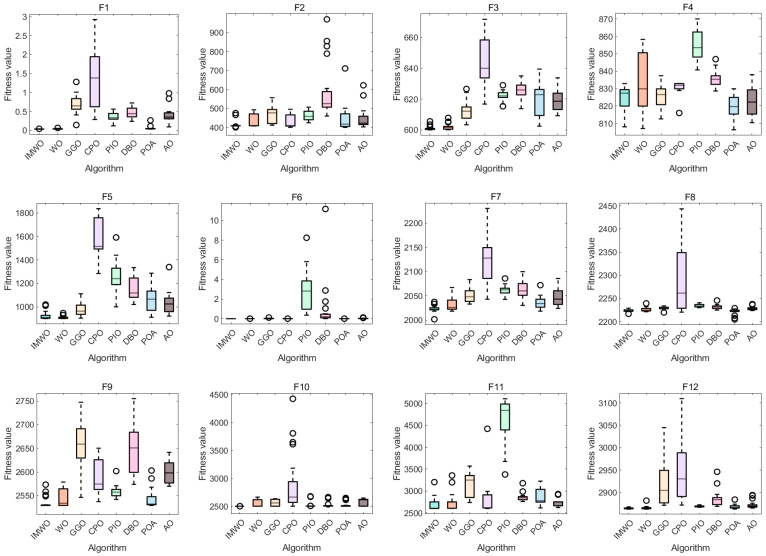
Boxplot comparison of various algorithms on CEC2022 functions.

Figure 7 displays the fitness values of eight algorithms across 12 different functions. As can be clearly observed from the figure, the IMWO algorithm outperforms the original algorithm WO on most functions; its median fitness values and overall distribution characteristics are consistently superior, indicating that the proposed improvement measures have effectively enhanced the algorithm’s performance. Other comparative algorithms exhibit varying performances across different functions. For instance, POA demonstrates good performance on functions such as F4 and F8, but its overall stability is inferior to that of IMWO. There are notable differences in the advantages of different algorithms across various functions, with no single algorithm performing optimally on all functions. IMWO demonstrates strong overall competitiveness, which fully validates the effectiveness and robustness of the proposed improvement strategies.

**Figure 8:**
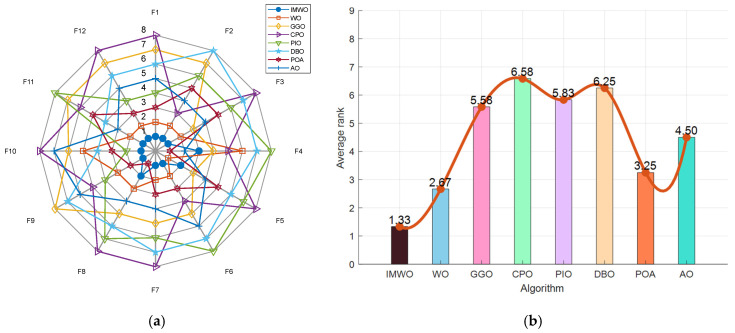
(**a**) Radar chart of various algorithms on CEC2022 functions; (**b**) ranking chart of various algorithms on CEC2022 functions.

Figure 8 presents a comprehensive performance comparison of competing algorithms on the CEC2022 benchmark functions. Figure 8a is a radar chart, where lines of different colors represent the performance of different algorithms across multiple functions; the closer that line is to the center, the better the algorithm’s performance. It can be observed that IMWO performs outstandingly on most functions. Figure 8b combines a bar chart and a line chart to present the average ranking of each algorithm, with lower values indicating better performance. The average ranking for WO is 2.67, while that for IMWO is 1.33; this represents a significant performance improvement over WO, confirming that the proposed improvement strategies effectively enhance the algorithm’s optimization capability. Other algorithms, such as CPO, with an average ranking of 6.58, perform poorly. POA ranks 3.25, and AO ranks 4.50, both lagging behind IMWO in overall performance. This demonstrates that IMWO outperforms WO and other compared algorithms on the CEC2022 benchmark functions, achieving better results across multiple functions and leading in average performance ranking, which reflects the effectiveness of the improvement measures. For 20-dimensional optimization problems, the comprehensive performance of the IMWO algorithm outperforms that of the WO algorithm and other comparative algorithms. Relevant detailed statistical data can be found in Figures S5 and S6 of the Supplementary Material.

## 5. Application of IMWO Algorithm in Coverage Optimization of WSNs

### 5.1. WSN Coverage Model

To simplify the mathematical optimization model of the objective function, this study adopts the Boolean perception model as the node sensing model for the WSN coverage optimization problem in a two-dimensional plane. The WSN Boolean sensing coverage model is an important model used in WSNs to describe the sensing capability of sensor nodes and regional coverage, characterized by its simplicity and intuitive expression. In this model, the sensing range of a sensor node is a circular area centered at the node with a sensing radius as the radius. Only target points that fall within this area are considered to be covered by the node, exhibiting a clear binary characteristic of “yes” or “no”; hence, it is also known as the 0/1 sensing model.

Assuming that the WSN monitoring area is a two-dimensional plane, the monitoring area is digitized into an *L* × *M* grid, with each grid cell considered as a target point. There are *N* homogeneous sensor nodes deployed, denoted as a set $Z=\left\{Z_{1},Z_{2},\ldots ,Z_{N}\right\}$. The coordinates of the node $Z_{i}$ are $\left(x_{i},y_{i}\right)$, and the coordinates of the target point $S_{j}$ are $\left(x_{j},y_{j}\right)$. According to the distance formula between two points in a two-dimensional space, the distance between node $Z_{i}$ and target point $S_{j}$ is given by formula (17), which is used to determine whether the target point is within the sensing range of the node.

(17) $$d(Z_{i},S_{j})=\sqrt{{\left(x_{i}-x_{j}\right)}^{2}+{\left(y_{i}-y_{j}\right)}^{2}}$$

$P(Z_{i},S_{j})$ is used to represent the perception reliability of node $Z_{i}$ towards the target point $S_{j}$. If $d(Z_{i},S_{j})\leq R_{s}$, then $P(Z_{i},S_{j})=1$, indicating that the target point can be effectively perceived by the node; otherwise, $P(Z_{i},S_{j})=0$, indicating that the target point cannot be perceived by the node.

To improve the probability of target perception, multiple sensors often need to collaborate for detection. The joint perception probability of a WSN for the target point $S_{j}$ is $Q(Z,S_{j})$, calculated using formula (18). Based on the perception quality of each node towards the target point, the perception probability of the target point under the combined effect of multiple nodes can be calculated.

(18) $$Q(Z,S_{j})=1-\prod_{i=1}^{N}\left(1-P(Z_{i},S_{j})\right)$$

Coverage is an important indicator for measuring the coverage performance of WSNs, denoted by *COV*. Coverage is defined as the ratio of the number of target points covered by all sensor nodes to the total number of target points in the area, and the coverage *COV* formula is shown in (19). A higher coverage indicates more effective coverage of the target area by the network, enabling more comprehensive monitoring of information within the target region.

(19) $$COV=\frac{\sum_{j=1}^{L\times M}Q(Z,S_{j})}{L\times M}$$

### 5.2. Mathematical Formulation of WSN Coverage Optimization Problem

To reformulate the WSN coverage optimization problem into a form amenable to solution by intelligent optimization algorithms, this section establishes a comprehensive mathematical model and elaborates on its encoding scheme, constraint handling method, and discretization strategy.

#### 5.2.1. Objective Function

Under the Boolean sensing model, the coverage optimization problem can be formulated as maximizing the coverage rate of the monitored area. To conform to the common formulation of optimization algorithms (i.e., minimizing the objective function), the problem is transformed into minimizing the uncovered rate, and the objective function is given by Equation (20).

(20) $$\min f(X)=1-COV=1-\frac{\sum_{j=1}^{L\times M}Q(Z,S_{j})}{L\times M}$$

where *f*(*X*) denotes the uncovered rate, which is the fitness function to be minimized.

#### 5.2.2. Constraint Conditions

The deployment of sensor nodes must satisfy the boundary constraints of the monitored area; that is, the node coordinates must fall within the range of the area. The constraint conditions are given by Equation (21).

(21) $$1\leq x_{i}\leq L,\;1\leq y_{i}\leq M,\;i=1,2,\ldots ,N$$

#### 5.2.3. Individual Encoding Scheme

In the optimization algorithm, each individual is directly encoded as a real-valued vector of length 2*N*. The individual encoding is given by Equation (22).

(22) $$X=[x_{1},x_{2},\ldots x_{N},y_{1},y_{2},\ldots y_{N}]$$

where $x_{i}\in [1,L]$ and $y_{i}\in [1,M]$ denote the abscissa and ordinate of the *i-th* node, respectively.

#### 5.2.4. Constraint Handling Technique

For boundary constraint handling, the boundary truncation method is adopted. If the coordinate values in the encoding vector exceed the range of [1, *L*] or [1, *M*], they are truncated to the corresponding boundary values.

(1) If $x_{i}<1$, set $x_{i}=1$. If $x_{i}>L$, set $x_{i}=L$
(2) If $y_{i}<1$, set $y_{i}=1$. If $y_{i}>M$, set $y_{i}=M$.

#### 5.2.5. Discretization of Continuous Optimization Algorithms

Because the monitored area has been digitized into an *L* × *M* grid (where the target points have integer coordinates), the positioning of sensor nodes must correspond to integer grid points. For optimization algorithms that output continuous values, a rounding operation is adopted to achieve discretization. After the algorithm outputs the continuous coordinate vector *X*, the floor function is applied to each coordinate to convert the continuous values into integers.

### 5.3. WSNs Coverage Experiment

To verify the performance of the IMWO algorithm in WSN coverage optimization, it was compared with seven other algorithms: WO, GGO, CPO, PIO, DBO, POA, and AO. Two sets of experiments were conducted with parameter adjustments. The experiments were simulated using MATLAB R2024a.

#### 5.3.1. Environment 1

**Figure 9:**
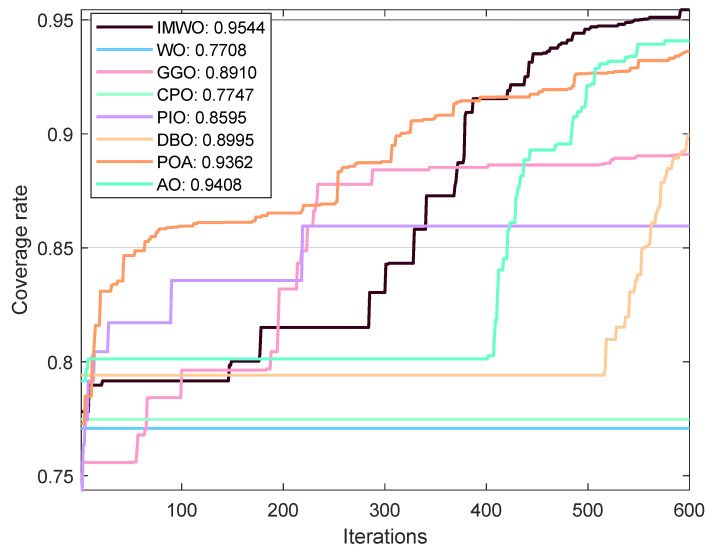
Comparison of algorithm coverage rates in the 30th run (Environment 1).

**Figure 10:**
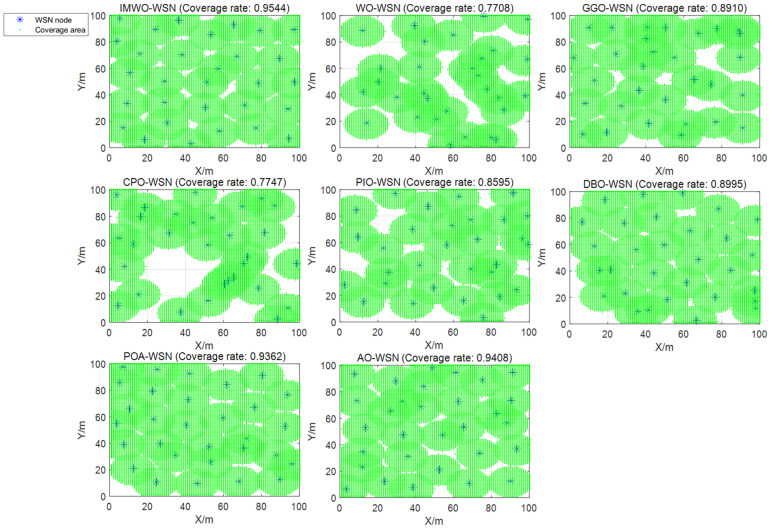
Coverage optimization comparison chart for the 30th run (Environment 1).

**Figure 11:**
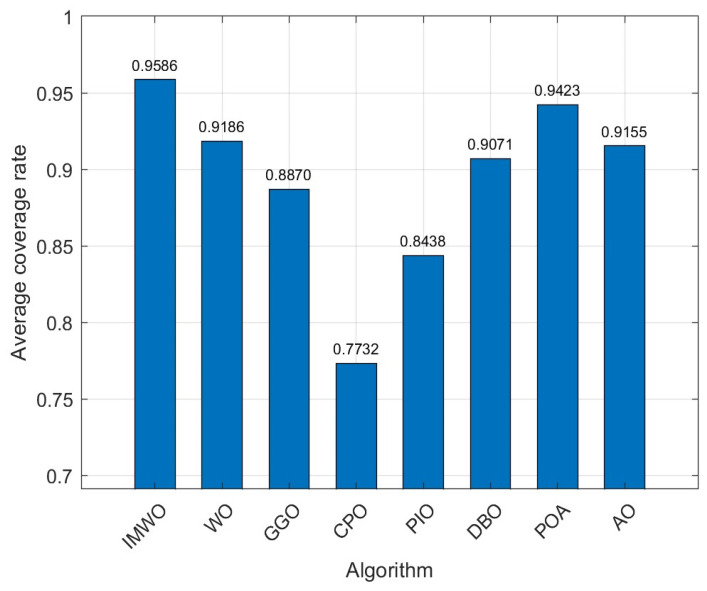
Bar chart of average coverage rate from 30 runs (Environment 1).

The simulation environment consists of a 100 × 100 monitoring area, where a Boolean communication model is adopted. The number of sensor nodes N=30, the sensing radius $R_{s}=6$, and the communication radius $R_{c}=12$. The population size for all algorithms is set to 30, with a maximum iteration count of 600. To avoid result bias due to experimental randomness, each algorithm is independently run 30 times, and the average value of the experiments is taken as the final result. The coverage curve for the 30th run is shown in Figure 9, and the coverage optimization distribution comparison of the 30th run is shown in Figure 10. As can be seen from Figure 9 and Figure 10, the node distribution of IMWO in a single run is more uniform and it achieves the highest coverage rate, surpassing the original algorithm WO and the other six comparison algorithms. The average coverage results of the eight algorithms after 30 operations are shown in Table 5 and Figure 11. It can be seen from Table 5 and Figure 11 that IMWO’s average value of 0.9586 is the highest among all algorithms, exceeding WO’s 0.9186. The maximum value of 0.9688 is also the best, higher than the 0.9605 of POA and 0.9439 of AO. The minimum value of 0.9454 is also the highest, and far higher than the 0.7635 of WO. IMWO has obvious advantages over WO, with its average performance being 4.0 percentage points higher and maximum coverage rate 0.88 percentage points higher, while the minimum value leads by 18.19 percentage points. This indicates that IMWO not only performs better overall, but also shows a significant improvement in stability.

#### 5.3.2. Environment 2

**Figure 12:**
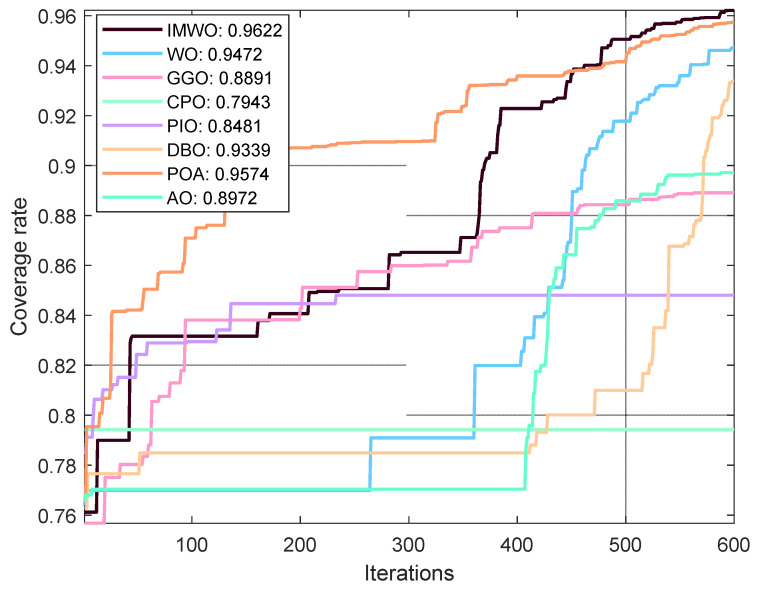
Comparison of algorithm coverage rates in the 30th run (Environment 2).

**Figure 13:**
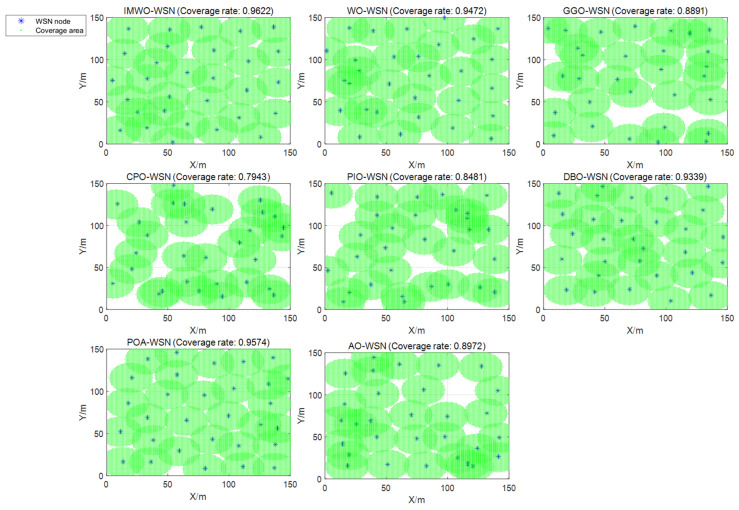
Coverage optimization comparison chart for the 30th run (Environment 2).

**Figure 14:**
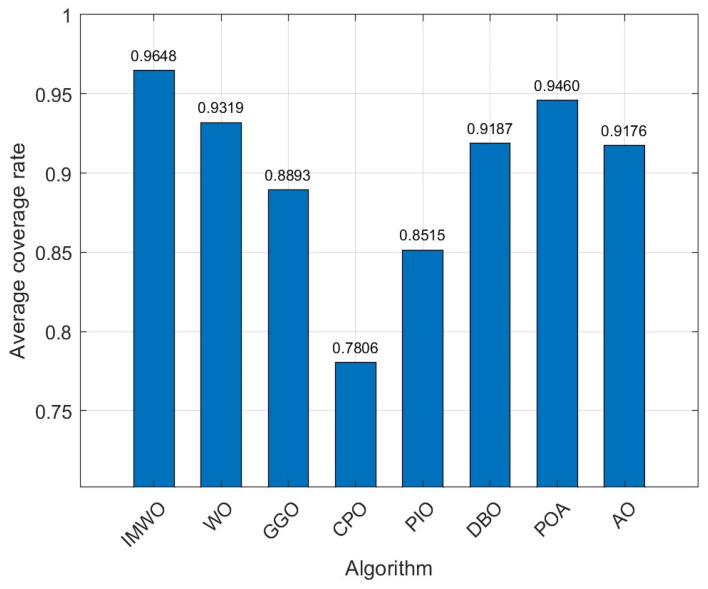
Bar chart of average coverage rate from 30 runs (Environment 2).

The simulation environment consists of a 150 × 150 monitoring area, where a Boolean communication model is adopted. The number of sensor nodes N=30, the sensing radius $R_{s}=9$, and the communication radius $R_{c}=18$. The population size for all algorithms is set to 30, with a maximum iteration count of 600. Similarly, each algorithm is independently run 30 times, and the average value of the experiments is taken as the final result. The coverage curve for the 30th run is shown in Figure 12, and the comparison of the coverage optimization distribution for the 30th run is shown in Figure 13. As can be seen from Figure 12 and Figure 13, the node distribution of IMWO in a single run is more uniform, and it achieves the highest coverage rate, surpassing the original algorithm WO and the other six comparison algorithms. The average coverage results of the eight algorithms after 30 runs are shown in Table 6 and Figure 14. As can be seen from Table 6 and Figure 14, the IMWO algorithm demonstrates significant advantages. Its average score is 0.9648, which is higher than that of all the other algorithms, such as WO’s 0.9319 and GGO’s 0.8893. IMWO exhibits the best stability and overall performance. Its maximum value of IMWO is 0.9803, and the minimum value is 0.9464, both of which are the highest among all algorithms, with minimal fluctuation (the difference between the maximum and minimum values is only 0.0339). This is far superior to WO’s fluctuation of 0.1831, indicating that IMWO maintains high efficiency and stability across different scenarios. Compared to the original WO algorithm, IMWO not only improves the average performance by approximately 3.29% but also addresses the drawback of WO’s excessively low minimum value, making it more reliable in extreme cases. The comprehensive advantages of IMWO are evident.

### 5.4. Feasibility Analysis of Node Deployment

The node layout scheme obtained by the IMWO algorithm in WSN coverage optimization exhibits excellent deployment feasibility in engineering practice, mainly reflected in the following aspects:

(1) Feasibility of node positions

All node coordinates optimized by the IMWO algorithm fall within the scope of the two-dimensional monitoring area without overlap or boundary exceeding. In actual deployment, sensor nodes can be accurately placed via GPS positioning or ground marking, which complies with the spatial constraints of practical deployment.

(2) Guarantee of communication connectivity

The communication radius $R_{c}$ and sensing radius $R_{s}$ adopted in the experiment both meet the common connectivity conditions of $R_{c}\geq 2R_{s}$, ensuring that the optimized network maintains global connectivity while achieving coverage optimization. This avoids isolated nodes and satisfies the networking requirements of practical WSNs.

(3) Considerations for energy consumption and deployment cost

By optimizing coverage to reduce redundant nodes, the IMWO algorithm minimizes the number of nodes while ensuring a good coverage rate, thereby lowering deployment costs and long-term maintenance energy consumption. The uniform distribution of nodes avoids regional energy hotspots, which helps extend the network lifetime.

## 6. Conclusions and Future Work

In summary, based on a combined strategy of DE/best/1 mutation, LSC Mapping, and Beta-OBL, this study proposes the IMWO algorithm, which aims to address the shortcomings of the WO algorithm, including its tendency to become trapped in local optima, insufficient stability, and inadequate population diversity. The experimental results show that, in CEC2017 and CEC2022 standard function tests, IMWO achieves average rankings of 1.66 and 1.33, ranking first among eight compared algorithms, outperforming WO’s rankings of 3.17 and 2.67; the IMWO algorithm exhibits smaller performance fluctuations and stronger stability. In WSN coverage optimization, IMWO’s average coverage rates are 95.86% and 96.48% in two experiments, compared with WO’s 91.86% and 93.19%; the IMWO algorithm achieves a higher coverage rate. However, the algorithm still faces challenges: its convergence speed significantly decreases in ultra-high-dimensional optimization scenarios. Furthermore, in dynamic problems where the solution space changes over time, it lacks the capability of real-time adaptive adjustment in search strategies, leading to performance degradation.

Subsequent research will explore hybrid strategies combining IMWO with other intelligent optimization algorithms. By integrating the search advantages of different algorithms, we aim to further enhance global optimization capabilities and improve the algorithm’s stability in complex scenarios. Meanwhile, the algorithm will be extended to fields such as energy-efficient routing in WSNs and complex engineering design to verify its universality, reduce application costs and resource consumption, and provide efficient optimization solutions for engineering problems.

## Figures and Tables

**Table 1:** Performance comparison of various algorithms on unimodal functions F1 and F3 and simple multimodal functions F4–F10.

F	Results	IMWO	WO	GGO	CPO	PIO	DBO	POA	AO
F1	min	229.5899	1514.1104	10110687.57	1240.5765	271163486	565880212.4	76023.2086	299262.5395
	std	5502.0663	76485.6298	1026410275	3149.0295	285347740	1116835479	343198165.5	8643719.105
	avg	6120.8922	54625.2264	593689682	**5023.0217**	751810422.7	2021031126	151397289.5	5376114.64
	median	4561.0063	17709.7228	195560412.5	4861.328	715005873.9	1816216024	6094448.793	2668760.003
	worse	19609.1082	267807.7199	4576118242	15372.0919	1258329744	4322879855	1447695900	39599068.44
F3	min	300.5217	321.7214	499.2726	396.0301	1228.4376	764.9891	302.5064	437.0015
	std	80.9322	172.3206	2667.4569	689.5262	1775.6208	1526.963	951.7175	831.6011
	avg	**345.9616**	461.9625	4188.458	1182.5837	4365.4168	2971.3503	865.5925	2034.6042
	median	319.5065	417.7624	3796.1003	1055.3883	4449.8037	2692.4853	485.682	1942.4904
	worse	661.9365	1108.8232	10224.8966	3241.3929	8164.689	6491.3006	3936.2471	4226.7034
F4	min	403.0676	406.4399	408.4511	400.1846	418.045	444.1223	400.2534	405.7822
	std	15.4313	31.46	59.4613	28.1295	25.5328	53.503	28.9049	29.3232
	avg	**411.8398**	422.9193	481.5752	421.2145	449.7287	516.417	418.4694	430.2193
	median	407.8287	408.1152	475.8696	407.5816	443.1113	499.3778	407.7155	410.2051
	worse	471.4807	506.3395	636.3441	477.5156	523.2452	615.8136	510.0154	494.7409
F5	min	511.9395	507.0788	511.9397	517.9136	539.5475	533.7721	518.0556	512.5957
	std	9.06	18.9687	7.3946	29.1229	6.046	10.1632	14.4469	12.0387
	avg	**521.7371**	530.989	525.1663	570.3853	552.7717	555.0003	541.9866	532.9793
	median	518.4093	522.4507	524.0469	569.6486	553.6671	554.2658	539.5077	533.2233
	worse	542.3019	568.7045	538.6793	632.3305	566.9499	578.5985	578.1521	561.4106
F6	min	600.064	600.1256	603.7472	630.0116	615.0625	615.6831	611.3747	607.0817
	std	1.6015	1.8061	5.5981	13.7649	4.417	4.3212	8.4339	6.1913
	avg	**601.2462**	601.4716	611.3701	653.5707	621.3962	624.7477	622.2958	618.4894
	median	600.5418	600.9138	609.7271	658.0198	620.4371	624.6354	622.7134	618.3326
	worse	605.4175	608.239	623.8229	682.827	635.0634	634.4149	638.9088	632.8283
F7	min	718.7804	719.3968	732.982	744.1282	796.8374	768.1144	739.4003	726.5341
	std	13.5327	24.4498	10.7231	30.9305	12.9031	11.5384	19.1601	14.0051
	avg	**735.6147**	745.4903	752.5627	795.7616	825.6774	792.7125	768.5038	752.9987
	median	733.6222	738.4014	749.1514	808.6529	825.2444	796.2198	768.8613	754.2325
	worse	767.7707	800.5921	771.5405	838.5385	846.4149	815.0133	805.6343	780.0947
F8	min	809.9496	809.9724	813.529	829.8504	851.6821	824.272	811.9712	812.4217
	std	7.6476	15.5476	9.1514	8.2309	5.0561	7.6949	7.0386	6.9384
	avg	**820.8548**	827.2489	829.7674	836.2229	861.0853	837.7	822.101	825.6821
	median	819.9042	821.4297	829.397	834.8244	860.3997	840.0864	822.8976	826.1937
	worse	835.7039	861.9793	845.7413	868.7378	868.6984	851.9244	834.8741	845.239
F9	min	900.0022	900.0503	916.4928	1247.9379	1080.5072	1005.9067	912.7557	917.3138
	std	105.1594	2.7882	96.5387	240.9497	133.1309	81.2858	110.2729	115.4174
	avg	926.2266	**902.4348**	1021.0918	1757.0607	1295.7673	1097.2725	1063.7486	1047.0945
	median	900.8962	901.7309	995.1949	1778.2655	1248.349	1082.9338	1035.995	1003.8728
	worse	1372.0391	911.8626	1258.9128	2215.2321	1576.2845	1282.3995	1283.4517	1385.8568
F10	min	1252.0799	1277.0561	1379.2380	1624.1917	2207.1308	1805.6469	1147.5864	1241.2476
	std	224.127	492.2836	333.2166	689.4722	150.1691	252.9751	227.9144	356.083
	avg	**1618.1277**	2052.2398	2198.063	2745.8701	2552.9155	2242.751	1652.2728	1992.3633
	median	1610.7231	2090.3861	2152.9187	2716.5655	2578.1971	2201.9813	1661.9111	1944.8884
	worse	1985.5976	2771.2539	2852.308	3962.6892	2882.8640	2638.8492	2011.1263	2640.1488

**Table 2:** Performance comparison of various algorithms on hybrid functions F11–F20.

F	Results	IMWO	WO	GGO	CPO	PIO	DBO	POA	AO
F11	min	1105.0401	1106.4845	1134.2051	1111.1286	1158.5683	1177.8496	1104.0149	1137.394
	std	7.4677	46.6343	68.3782	78.1098	52.9441	131.2581	23.296	64.6975
	avg	**1118.7062**	1126.822	1214.9557	1210.7824	1247.0935	1319.6013	1143.8466	1221.8512
	median	1117.5289	1117.002	1189.657	1170.3375	1237.6253	1302.7773	1139.9236	1204.5332
	worse	1134.5478	1322.2406	1384.2862	1342.9682	1363.0518	1681.9231	1194.2583	1371.6339
F12	min	27348.2573	46356.8737	235846.2142	14407.1234	4454153.385	374046.7916	3936.766	25087.7879
	std	1087639.39	2292405.31	3778282.218	3719510.919	9500037.684	6014236.701	469267.1119	5155460.59
	avg	1032929.826	1619650.446	3975088.978	3214690.844	22725738.81	6535394.385	**267695.6851**	4935249.927
	median	755841.533	856456.0452	2517387.272	1822050.197	21151447.95	5552206.158	55065.3421	3301671.905
	worse	3153999.856	9682730.728	13357573.41	13542585.66	37679991.6	21545629.34	1915631.875	18742703.11
F13	min	1738.0868	1639.6599	3901.6542	2151.2783	2485.758	3122.2449	1421.4545	3145.4109
	std	8129.737	10534.1809	10455.0292	10887.4728	112060.6236	16426.2679	875.6345	10265.1175
	avg	9067.0123	11264.8391	18848.5963	14693.0921	111505.8829	21316.3868	**2178.2663**	16835.5751
	median	7048.52	7806.2571	17449.9877	10976.6055	85699.7064	16966.7112	1881.7846	14929.2067
	worse	31673.1223	36324.43	38565.4355	39527.3009	406801.1876	66799.2464	5038.1004	44257.0383
F14	min	1460.87	1491.707	1478.6913	1518.181	1484.5997	1593.6817	1428.7963	1532.2278
	std	357.3378	753.867	348.7142	4380.5527	123.1417	1507.29	24.4006	686.1676
	avg	1665.4103	2085.4354	1638.4712	5824.1583	1583.3582	2479.0467	**1459.9696**	2159.1357
	median	1526.5996	1665.9745	1537.7864	3749.5806	1539.4262	1933.4026	1457.4682	1744.1782
	worse	3028.5281	4035.3537	3089.9913	15169.0442	1924.0547	8042.3526	1519.4066	3597.89
F15	min	1586.8966	2088.6562	1733.3396	5306.1325	1729.3479	2075.993	1540.5918	2029.2618
	std	1778.5408	2181.5293	3860.1511	25471.5447	1953.6893	2109.7022	113.5622	3533.8271
	avg	2933.8422	5367.5377	5769.2982	37374.9136	4207.6732	5017.7336	**1669.6391**	7203.601
	median	1950.533	4944.7671	4190.0381	34875.7235	3833.9456	4339.2903	1629.471	7638.7217
	worse	7731.7203	8926.7683	16161.7631	115410.6399	10713.2053	9328.2343	1989.8143	12303.0979
F16	min	1601.9363	1604.6934	1611.4743	1772.6597	1621.2325	1631.6925	1603.5087	1628.6066
	std	57.0825	135.0853	129.6932	214.2007	114.0101	119.8398	145.8043	117.0683
	avg	**1663.9656**	1734.3802	1802.2584	2147.2688	1804.0806	1795.3071	1831.5789	1817.0119
	median	1641.9959	1684.4158	1768.8926	2234.8145	1794.3730	1758.3041	1867.8676	1836.5927
	worse	1735.4308	2039.9912	2028.1663	2603.5273	1985.7132	1989.1771	2022.5763	2068.2059
F17	min	1719.2855	1723.1386	1736.607	1752.4241	1765.3248	1754.8302	1738.7865	1748.5383
	std	10.7317	37.3888	23.1437	122.3966	33.2194	17.3231	14.9408	23.4107
	avg	**1742.3737**	1756.5263	1769.4125	1862.2362	1821.722	1788.2489	1755.6989	1783.482
	median	1741.7783	1744.7684	1765.0509	1810.6485	1820.495	1786.487	1753.1802	1776.4348
	worse	1766.1177	1889.353	1823.6559	2140.8712	1894.2990	1838.4555	1800.5759	1832.8222
F18	min	2382.7584	4001.1033	3876.9439	3144.1132	8183.2613	12349.5497	1873.2937	6594.704
	std	12327.8138	13419.6588	12593.6305	13079.3196	221604.0525	63870.4902	767.6190	29043.4261
	avg	13699.9839	21548.9352	18531.993	18447.4066	185126.9432	62538.3361	**2225.2048**	34108.2616
	median	9639.1175	17029.1431	17230.763	15288.3768	122130.8303	45415.189	1991.4102	30008.1689
	worse	51091.5429	48990.6192	51885.6925	44616.3889	987679.913	277304.6069	5338.0863	144154.2336
F19	min	1973.0025	2021.1802	1949.4797	3845.5165	2018.3083	2012.1923	1907.2203	2062.3777
	std	3753.6467	8587.3817	34360.957	32875.807	3531.3444	12346.5466	38.3979	141737.9888
	avg	4448.5361	9329.8431	27483.4556	26485.2768	4560.8931	7649.262	**1949.1307**	58059.5065
	median	3094.3688	5356.646	21244.7603	17978.6866	3041.5549	3243.2106	1935.0889	11388.2765
	worse	15919.8912	31064.9079	121488.0559	145236.8675	14917.2417	50845.4388	2048.3450	594439.9826
F20	min	2019.8032	2021.0816	2068.4260	2077.5557	2066.9307	2044.7820	2026.2547	2060.0220
	std	20.0532	68.9111	44.1455	105.2191	19.4115	45.3120	33.7308	65.5272
	avg	**2042.8015**	2085.7354	2150.4743	2278.0971	2103.9087	2121.8043	2082.8627	2141.9518
	median	2041.8704	2048.1312	2166.8253	2266.0354	2097.6742	2119.4272	2080.0821	2115.6644
	worse	2092.1450	2243.8174	2205.4786	2431.6926	2145.6650	2183.7730	2164.8623	2278.1432

**Table 3:** Performance comparison of various algorithms on composite functions F21–F30.

F	Results	IMWO	WO	GGO	CPO	PIO	DBO	POA	AO
F21	min	2200.1596	2201.718	2219.441	2204.1595	2205.8895	2207.381	2202.5577	2206.0188
	std	2.0400	53.5455	36.4030	46.0916	66.3857	9.2298	64.8794	53.6815
	avg	**2204.0445**	2235.2622	2315.9946	2347.9090	2311.0327	2219.5358	2276.5435	2296.7503
	median	2203.5811	2206.5858	2326.1501	2351.0024	2351.0413	2217.7989	2321.5631	2321.8994
	worse	2208.3737	2365.372	2346.8287	2406.3374	2362.5561	2245.0679	2353.8664	2347.8017
F22	min	2301.1031	2215.5847	2243.8515	2301.1598	2287.2857	2290.0009	2229.2989	2308.5467
	std	1.2285	20.1369	82.8172	367.5977	33.8895	86.7543	28.169	6.8472
	avg	2302.6325	**2300.1815**	2341.8255	2421.2724	2396.7279	2513.3258	2308.6197	2313.8048
	median	2302.1586	2303.4971	2321.1885	2305.9740	2400.3712	2515.5968	2306.0792	2312.2466
	worse	2305.2721	2313.3221	2628.6157	3754.5921	2438.724	2722.8209	2370.1653	2338.9287
F23	min	2300.6308	2611.024	2614.402	2628.7765	2633.0956	2644.8485	2622.0680	2621.1898
	std	99.689	14.6622	11.6307	73.9543	8.7141	10.8465	21.5246	12.3309
	avg	**2590.548**	2632.4079	2626.1043	2738.9535	2648.2192	2665.6717	2657.3092	2645.7939
	median	2617.7707	2628.7896	2622.1859	2729.0769	2648.0480	2665.2876	2656.9525	2644.7848
	worse	2645.5768	2657.0643	2657.0621	2879.4125	2666.1482	2686.8808	2704.1397	2667.918
F24	min	2500.0198	2500.706	2571.4055	2781.0533	2560.675	2556.3513	2506.2353	2504.2126
	std	112.7425	81.0674	57.4536	80.696	88.6265	71.5396	129.1594	97.8817
	avg	2692.7488	2731.9506	2741.19	2874.8179	2725.4865	**2656.4771**	2675.1178	2731.2863
	median	2751.5857	2747.1602	2759.1653	2855.596	2778.0051	2630.2587	2755.6799	2769.9843
	worse	2774.8976	2789.7076	2772.0234	3082.7767	2795.7638	2795.7126	2809.4017	2791.1542
F25	min	2898.8779	2899.4587	2950.2765	2898.6551	2960.4233	2944.3806	2899.194	2900.482
	std	19.1311	17.8332	42.0584	21.0318	14.7548	70.4064	30.7101	22.9014
	avg	2941.9876	2940.9713	2987.232	2931.1977	2980.0712	3030.5017	**2924.0063**	2936.3274
	median	2947.4762	2947.5951	2964.0683	2943.7890	2983.3165	3003.9651	2915.1157	2948.0836
	worse	2971.3606	2951.685	3099.199	2946.8005	3018.3035	3171.6076	3015.9233	2957.1229
F26	min	2800.2702	2800.516	2928.4773	2815.7108	2991.5522	2720.5126	2612.3271	2831.0166
	std	86.7297	89.8092	370.3839	479.2363	43.5265	150.6896	368.9634	166.8563
	avg	2964.1607	**2946.7829**	3246.3201	3793.5962	3052.3197	3196.5151	3046.1233	3119.2933
	median	2988.471	2990.0961	3091.2388	3863.6369	3043.0775	3196.1245	2971.7546	3094.1377
	worse	3095.6543	3034.4595	4057.6185	4619.823	3196.4277	3396.0498	4188.6439	3462.145
F27	min	3089.4108	3090.2111	3099.3919	3100.5343	3097.2323	3097.8991	3090.5951	3094.3498
	std	2.5029	2.9303	36.8838	94.0978	4.1821	9.8702	20.3212	11.8237
	avg	**3093.7957**	3094.3035	3139.9002	3198.2797	3102.7955	3113.0715	3106.4008	3109.8237
	median	3093.6628	3093.6124	3123.6658	3194.4469	3101.5158	3111.2795	3098.6977	3107.7196
	worse	3098.5364	3100.7389	3210.9413	3489.5337	3110.1368	3135.6846	3164.9049	3139.0721
F28	min	3100.0477	3101.7291	3148.1631	3171.942	3204.7144	3225.7018	3164.8254	3147.6581
	std	115.7014	106.1308	174.169	126.7377	71.0156	122.1159	108.4303	96.4327
	avg	**3258.253**	3343.2989	3527.943	3427.9638	3261.0895	3325.5935	3272.9912	3388.1083
	median	3208.6071	3407.2586	3540.6067	3417.9869	3233.4747	3273.7334	3223.2054	3414.324
	worse	3411.8266	3455.1398	3776.2388	3731.8129	3431.3271	3654.6227	3457.6925	3526.2244
F29	min	3164.4799	3158.4564	3176.0047	3210.5665	3177.2886	3183.5905	3138.5989	3171.1203
	std	37.9657	55.5159	75.7172	192.6548	47.3246	49.6229	59.1508	71.1785
	avg	**3205.1903**	3221.6957	3306.5231	3461.6191	3249.9584	3264.8044	3218.9208	3268.1494
	median	3199.264	3204.9638	3310.4156	3452.169	3235.9165	3257.8512	3213.8291	3257.996
	worse	3272.6218	3361.1913	3469.0587	3851.1552	3355.9257	3376.3874	3379.5762	3472.2724
F30	min	30252.59906	6213.65515	82215.02194	267512.7744	135508.9652	34862.08233	4315.279651	29057.08821
	std	428988.9833	568380.8789	2339630.326	39092962.95	1056614.115	843596.2531	649208.3027	1247700.773
	avg	483785.2761	638542.6962	2093586.084	14076373.83	1175731.91	1037324.466	**264744.0496**	1256386.523
	median	543179.61	613118.4805	1508209.003	2841227.959	627601.5527	673404.0011	24487.66634	667294.2432
	worse	1337783.626	1761182.427	10204750.19	177048702.6	4046653.238	2496848.854	2670235.021	3657021.434

**Table 4:** Performance comparison of various algorithms on CEC2022 functions.

F	Results	IMWO	WO	GGO	CPO	PIO	DBO	POA	AO
F1	min	301.2319	308.8127	1407.6323	2847.8952	1183.1228	2378.2323	326.6935	923.2229
	std	30.0508	79.1158	2430.2614	7886.3388	1069.1984	1441.3639	513.2846	2038.0502
	avg	**333.7049**	400.3915	6860.7324	13716.2728	3581.0344	4621.1677	563.2172	3957.3425
	median	325.4441	399.8951	6471.1067	13833.7499	3333.8556	4402.5659	418.2821	3487.0106
	worse	410.9764	642.2568	12779.4386	29256.5949	5607.0102	7228.9931	2634.1565	9775.7761
F2	min	400.1661	407.6442	411.5287	400.3287	424.5453	460.0716	400.1088	402.8532
	std	19.8356	33.2265	41.1527	35.6233	26.2288	144.951	70.5116	56.6608
	avg	**413.5831**	430.0402	467.9772	431.727	462.556	591.7204	447.4026	446.3143
	median	408.917	408.9525	476.8998	408.9487	458.1857	525.3774	417.1407	422.4101
	worse	475.4617	493.0827	556.8243	495.7052	506.7973	970.1802	711.1684	622.2135
F3	min	600.0385	600.0983	603.3525	616.8075	615.3295	613.8086	602.4643	609.2133
	std	1.3407	2.0503	6.2461	14.2728	3.0729	5.3278	10.3176	6.8029
	avg	**601.004**	601.9845	611.9256	644.0827	622.2025	625.5383	619.674	619.1271
	median	600.5784	601.6579	612.1653	640.0381	622.0826	625.9717	622.9509	618.6235
	worse	605.6112	607.8397	626.6052	671.8257	629.0297	635.0863	639.5044	633.8353
F4	min	807.962	807.0038	812.5257	815.9245	840.7054	828.579	806.2814	810.3431
	std	7.1341	18.1624	6.4763	3.7794	8.7992	4.858	6.1808	7.8419
	avg	824.8245	834.1466	825.8031	830.7508	854.7241	835.5376	**819.5077**	822.2671
	median	827.3614	829.8489	826.4992	831.8396	853.5553	835.35	819.6611	822.231
	worse	832.8337	858.2505	837.349	832.8971	870.0457	846.9005	829.8586	837.9829
F5	min	900.1791	900.1281	904.1184	1284.5214	1000.6013	1020.6565	911.7111	921.4213
	std	35.1529	11.7666	61.5948	169.5416	128.5494	92.1580	112.7116	96.0731
	avg	923.9841	**909.83**	980.0111	1587.5124	1260.2105	1152.1932	1070.8419	1028.8835
	median	909.6108	906.7935	963.5006	1514.9058	1240.0328	1117.8312	1065.3174	1024.7388
	worse	1019.7571	946.9283	1111.5718	1836.9933	1591.2445	1334.245	1286.5036	1338.875
F6	min	1873.0319	1892.9503	1933.8012	1996.6269	360888.7754	9780.4073	1839.1701	3064.4658
	std	1055.7919	1567.541	26796.363	1980.7364	2059366.715	2494344.107	1851.5800	29735.7878
	avg	**2719.865**	2923.1467	21885.0038	3567.6864	2938778.369	996113.4417	3074.0542	28722.8259
	median	2159.503	2083.7082	9678.124	2830.5633	2813182.175	216950.0427	2049.3317	18418.2677
	worse	5177.3711	7215.9152	114474.7114	8107.7765	8241636.616	11169295.27	8117.5537	125798.9876
F7	min	2001.6366	2018.2534	2032.894	2043.0266	2042.7065	2030.2868	2018.3855	2024.1328
	std	6.9452	13.957	14.7005	47.4043	9.685	16.9656	12.6998	18.444
	avg	**2023.1393**	2032.2102	2050.9843	2123.8751	2061.7201	2063.0778	2035.809	2047.7646
	median	2022.3547	2026.0084	2048.147	2128.0061	2062.4375	2060.2015	2034.1888	2043.1636
	worse	2037.4702	2067.0989	2083.651	2230.4202	2085.8826	2099.8667	2072.0595	2085.8293
F8	min	2216.8962	2221.3073	2219.7682	2220.489	2230.1868	2224.9471	2204.9386	2223.9326
	std	2.4066	4.5564	3.0395	72.5251	3.3091	4.5669	6.4073	3.5558
	avg	2223.7719	2226.3814	2229.0051	2290.6109	2234.6234	2231.9377	**2221.1605**	2228.5565
	median	2223.84	2224.4537	2229.5373	2261.7084	2233.7705	2231.4783	2222.8745	2227.489
	worse	2229.127	2239.3934	2233.9256	2443.4325	2240.785	2245.7019	2229.0651	2237.9449
F9	min	2529.2844	2529.2844	2546.4046	2537.2639	2542.0234	2573.9342	2529.3217	2569.9105
	std	12.5191	18.2577	51.6505	38.2704	13.5724	50.6865	20.5433	23.3337
	avg	**2535.1227**	2544.5091	2657.0235	2589.4996	2558.6611	2646.6758	2543.6152	2599.7523
	median	2529.2873	2533.8082	2658.8912	2574.498	2557.3166	2650.9184	2532.1945	2598.0737
	worse	2573.2953	2578.7141	2747.3665	2650.1719	2601.897	2755.0118	2603.0821	2641.5812
F10	min	2500.2923	2500.4582	2500.4645	2500.7316	2501.2197	2501.0440	2500.4713	2500.7794
	std	0.2074	66.1369	63.7310	545.4077	52.4380	53.2466	52.0713	57.6326
	avg	**2500.6858**	2557.9070	2562.8368	2892.0944	2520.3768	2529.0554	2527.0684	2586.4009
	median	2500.6844	2501.0850	2559.9584	2663.7499	2503.1791	2507.0302	2500.8270	2617.7033
	worse	2501.2473	2663.7307	2635.6511	4427.1392	2677.7296	2663.6162	2648.1179	2647.5973
F11	min	2600.3072	2602.3202	2737.0197	2600.4452	3377.7994	2758.4399	2612.5196	2622.2337
	std	148.1347	202.0257	287.7346	388.8202	484.7772	92.4758	194.2474	102.6919
	avg	**2728.8811**	2747.2780	3146.8392	2890.9026	4653.5451	2855.9118	2883.4671	2751.4343
	median	2750.5213	2746.8911	3249.2589	2910.2735	4842.5583	2843.5144	2777.1149	2751.0351
	worse	3202.9644	3354.7301	3570.9275	4422.6242	5109.0145	3178.1554	3226.0065	2930.2359
F12	min	2862.5674	2862.5674	2871.2209	2871.6328	2866.7451	2868.8950	2862.6965	2865.0492
	std	1.4903	4.1128	47.3316	70.2766	1.5162	18.5012	4.9476	8.7370
	avg	**2864.2695**	2865.5347	2916.4821	2948.4962	2868.8018	2885.7362	2868.0390	2872.1634
	median	2863.7596	2864.2938	2904.5743	2930.2209	2868.5332	2884.1021	2866.7111	2869.2006
	worse	2500.2923	2500.4582	2500.4645	2500.7316	2501.2197	2501.0440	2500.4713	2500.7794

**Table 5:** Statistical results of coverage rate from 30 runs (Environment 1).

Algorithm	IMWO	WO	GGO	CPO	PIO	DBO	POA	AO
average	0.9586	0.9186	0.8870	0.7732	0.8438	0.9071	0.9423	0.9155
maximum	0.9688	0.9600	0.9234	0.8102	0.8632	0.9440	0.9605	0.9439
minimum	0.9454	0.7635	0.8570	0.7411	0.8300	0.8720	0.9085	0.8721

**Table 6:** Statistical results of coverage rate from 30 runs (Environment 2).

Algorithm	IMWO	WO	GGO	CPO	PIO	DBO	POA	AO
average	0.9648	0.9319	0.8893	0.7806	0.8515	0.9187	0.9460	0.9176
maximum	0.9803	0.9712	0.9316	0.8220	0.8646	0.9501	0.9583	0.9420
minimum	0.9464	0.7881	0.8557	0.7517	0.8356	0.8755	0.9163	0.8907

## Data Availability

The original contributions presented in this study are included in the article/supplementary material. Further inquiries can be directed to the corresponding author.

## Supplementary Materials

The following supporting information can be downloaded at: https://www.mdpi.com/article/10.3390/biomimetics11010072/s1, Figure S1: Figure of convergence curve comparison of various algorithms on CEC2017 functions (30-dimensional); Figure S2: (a) Figure of radar chart of various algorithms on CEC2017 functions, (b) Figure of ranking chart of various algorithms on CEC2017 functions (30-dimensional); Figure S3: Figure of convergence curve comparison of various algorithms on CEC2017 functions (50-dimensional); Figure S4: (a) Figure of radar chart of various algorithms on CEC2017 functions, (b) Figure of ranking chart of various algorithms on CEC2017 functions (50-dimensional); Figure S5: Figure of convergence curve comparison of various algorithms on CEC2022 functions(20-dimensional); Figure S6: (a) Figure of radar chart of various algorithms on CEC2022 functions, (b) Figure of ranking chart of various algorithms on CEC2022 functions(20-dimensional).

## Abbreviations

WO	Walrus Optimization
IMWO	Improved Walrus Optimization
DE	Differential Evolution
LSC	Logistics–Sine–Cosine
Beta-OBL	Beta Opposition-Based Learning
WSNs	Wireless Sensor Networks
GGO	Greylag Goose Optimization
CPO	Chinese Pangolin Optimizer
PIO	Pigeon-inspired Optimization
DBO	Dung Beetle Optimizer
POA	Pelican Optimization Algorithm
AO	Aquila Optimizer
