# Supplementary material for: Multi-Strategy Fusion Improved Walrus Optimization Algorithm for Coverage Optimization in Wireless Sensor Networks
Source: Biomimetics (Basel). 2026 Jan 15;11(1):72. doi: 10.3390/biomimetics11010072 (PMC12838938; doi:10.3390/biomimetics11010072)
Supplement: Supplementary file 1 [file biomimetics-11-00072-s001.zip › biomimetics-4079731-supplementary.pdf]

# Supplementary Information

## 1. CEC2017 Test Functions（30-dimensional）

Figure S1 presents the convergence curve comparison results of various algorithms on CEC2017 test functions (30-dimensional), clearly demonstrating the significant advantages of the IMWO algorithm. From the overall trend, IMWO exhibits faster convergence speed and better convergence accuracy across most test functions: its curve often drops sharply in the early iterations and maintains a stable leading position in subsequent iterations, indicating that the algorithm has excellent ability to balance global exploration and local exploitation.

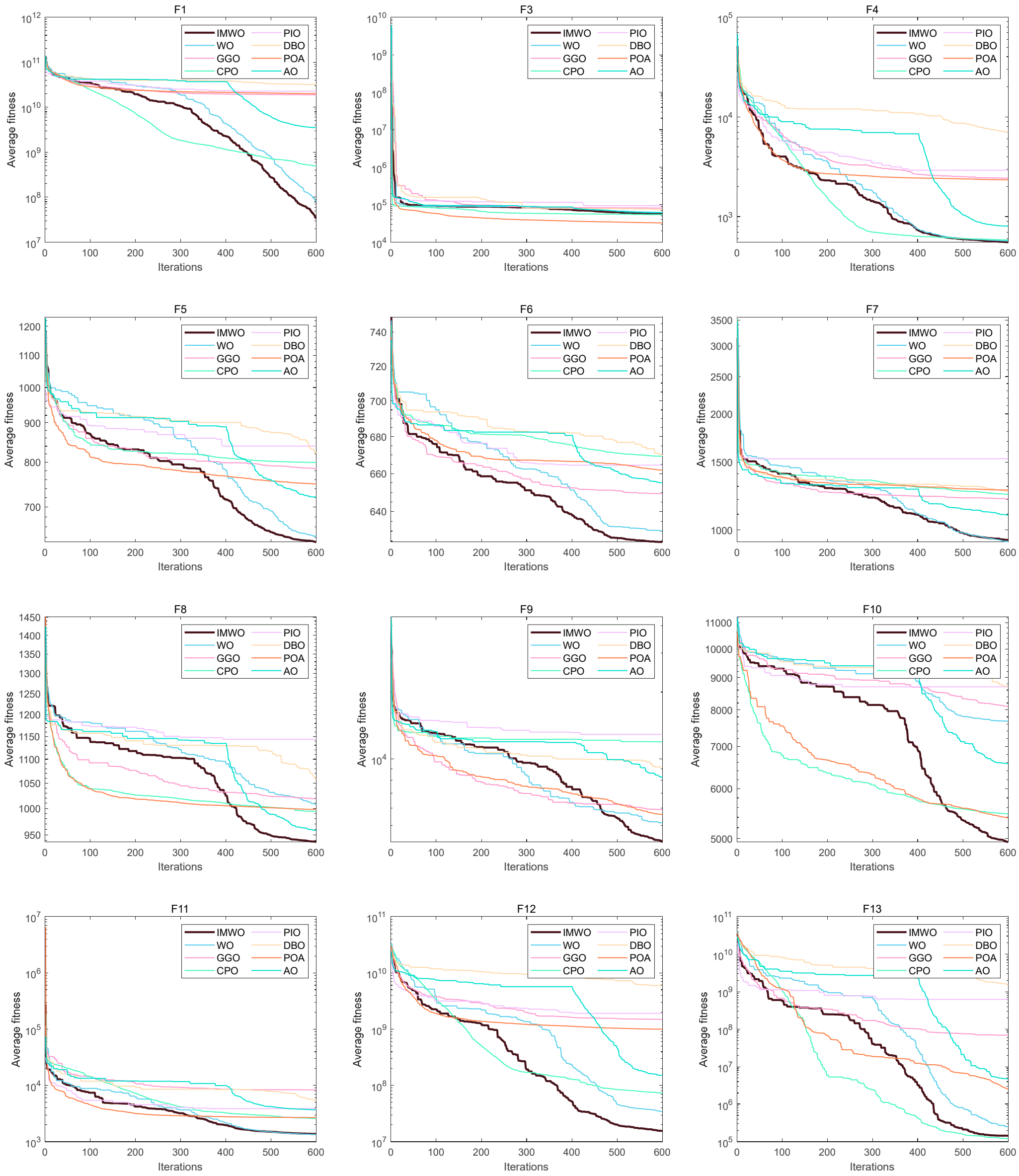

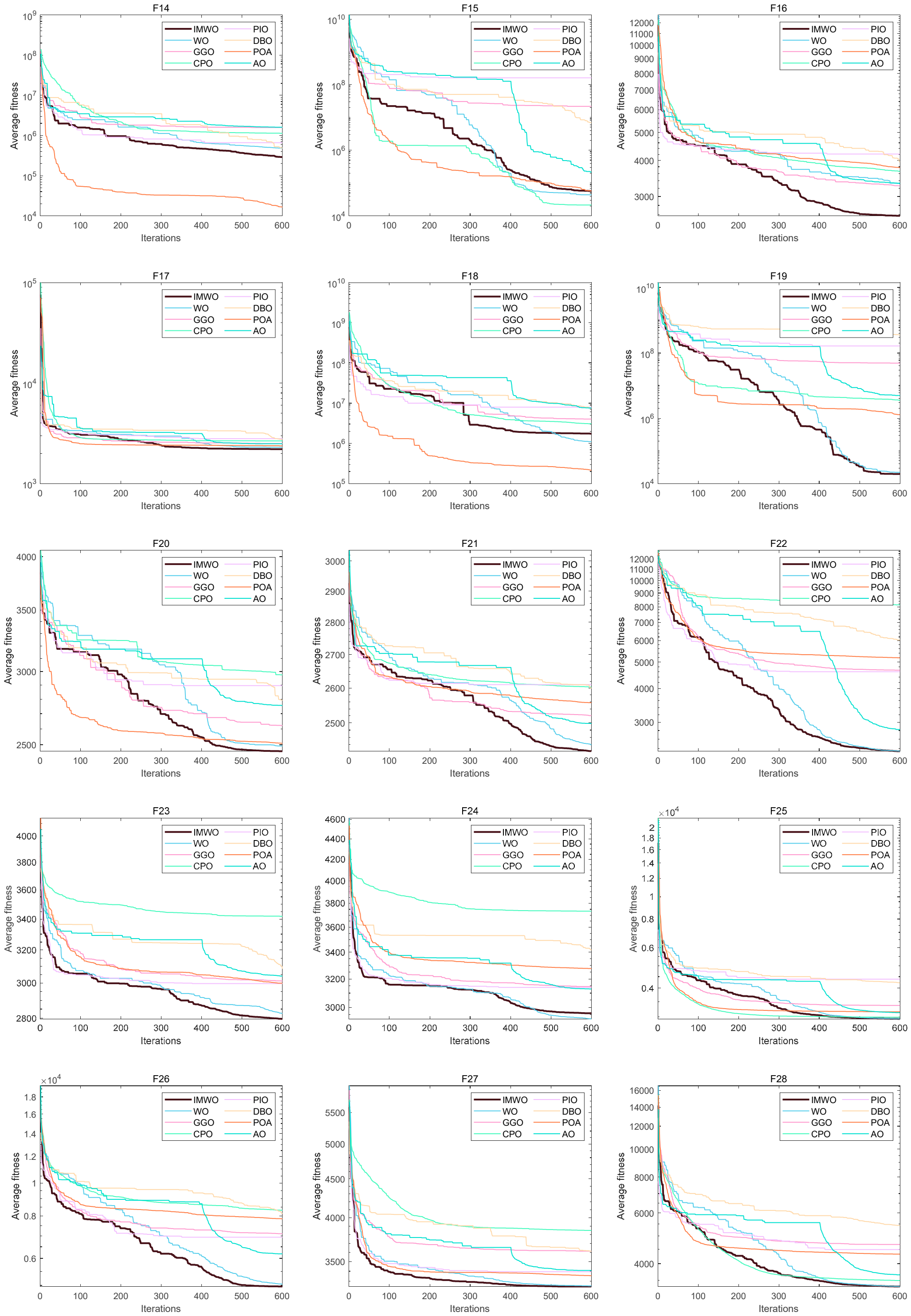
F14
F15
F16
F17
F18
F19
F20
F21
F22
F23
F24
F25
F26
F27
F28
Average fitness
Iterations
IMWO
WO
GGO
CPO
PIO
DBO
POA
AO

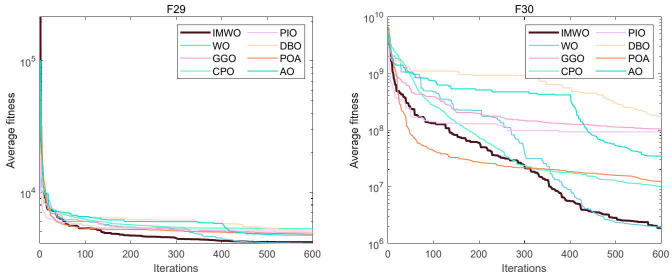


**Figure S1.** Figures of convergence curve comparison of various algorithms on CEC2017 functions (30-dimensional).

Figure S2 presents a comprehensive performance comparison of competing algorithms on the CEC2017 benchmark functions (30-dimensional). Figure S2(a) is a radar chart, where lines of different colors represent the performance of different algorithms across multiple functions; a line that is closer to the center indicates better algorithm performance. It can be observed that IMWO performs outstandingly on most functions. Figure S2(b) shows algorithms' average rankings (lower = better). IMWO's average ranking is 1.45, ranking first; WO's is 2.38, meaning IMWO outperforms WO significantly. Other algorithms (e.g., CPO with 5.45, POA with 4.07) lag far behind. This proves IMWO outperforms WO and other algorithms on 30-dimensional CEC2017 functions: it excels across most tasks and leads in average ranking, verifying the effectiveness of its improvement strategies.

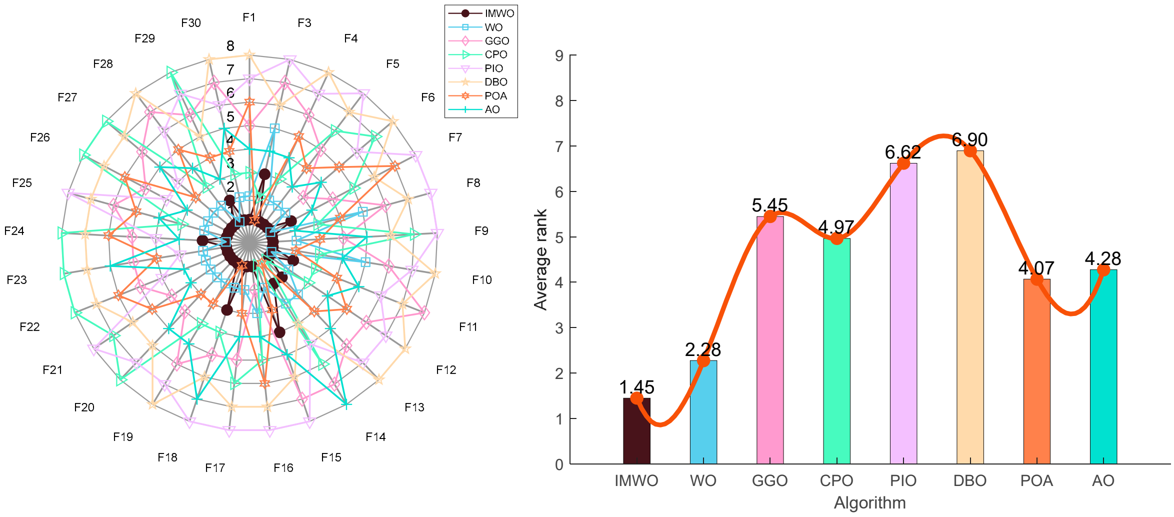


(a) (b)

**Figure S2.** (a) Figure of radar chart of various algorithms on CEC2017 functions, (b) Figure of ranking chart of various algorithms on CEC2017 functions (30-dimensional).

## 2. CEC2017 Test Functions（50-dimensional）

As shown in Figure S3, on the higher-dimensional CEC2017 test functions (50-dimensional), the advantages of the IMWO algorithm are further highlighted. Its convergence curves consistently maintain a

steep downward trend in the early iterations across multiple test problems, and finally stabilize at significantly lower fitness values—indicating that the algorithm can effectively handle the optimization complexity brought by increased dimensionality.

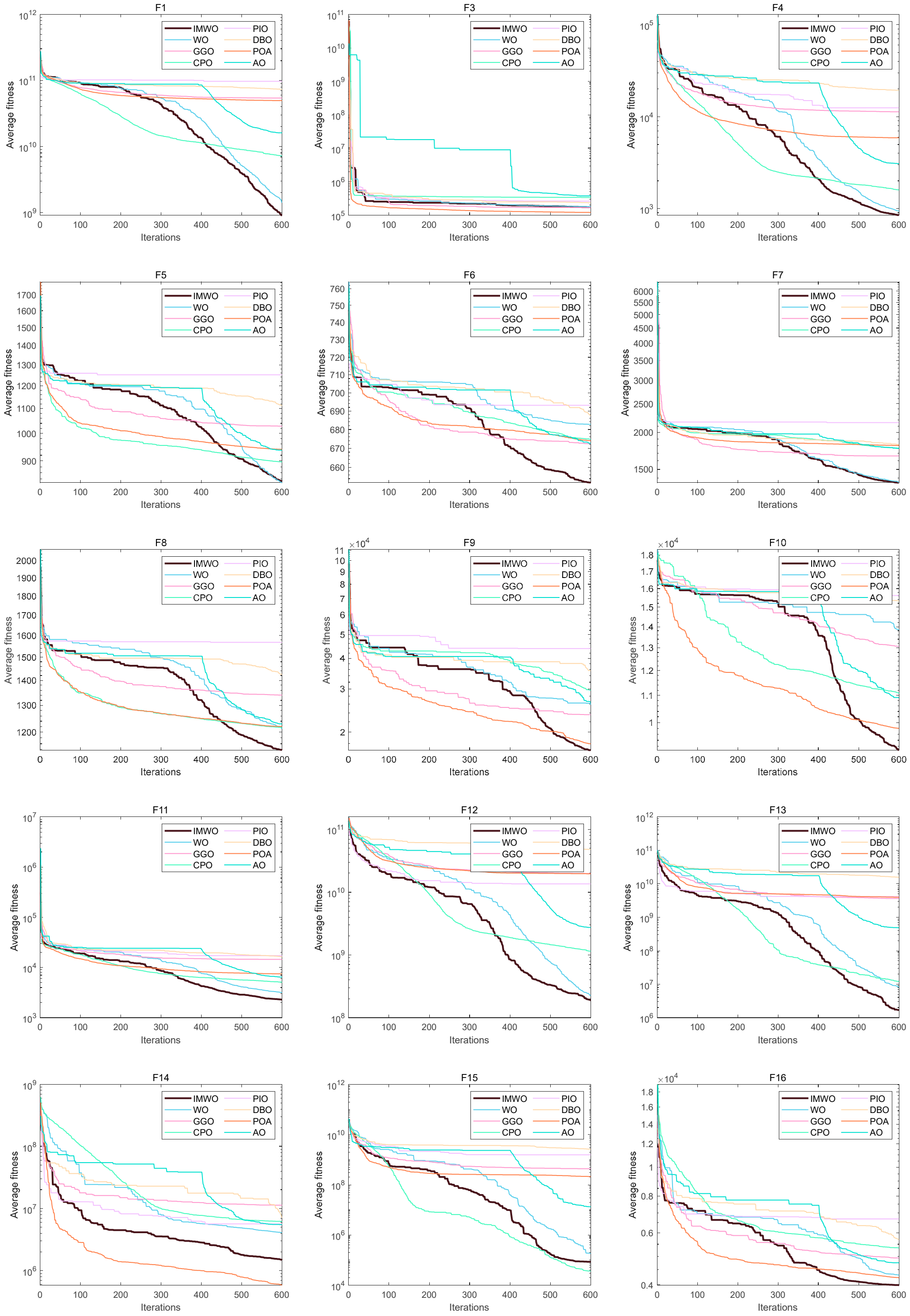

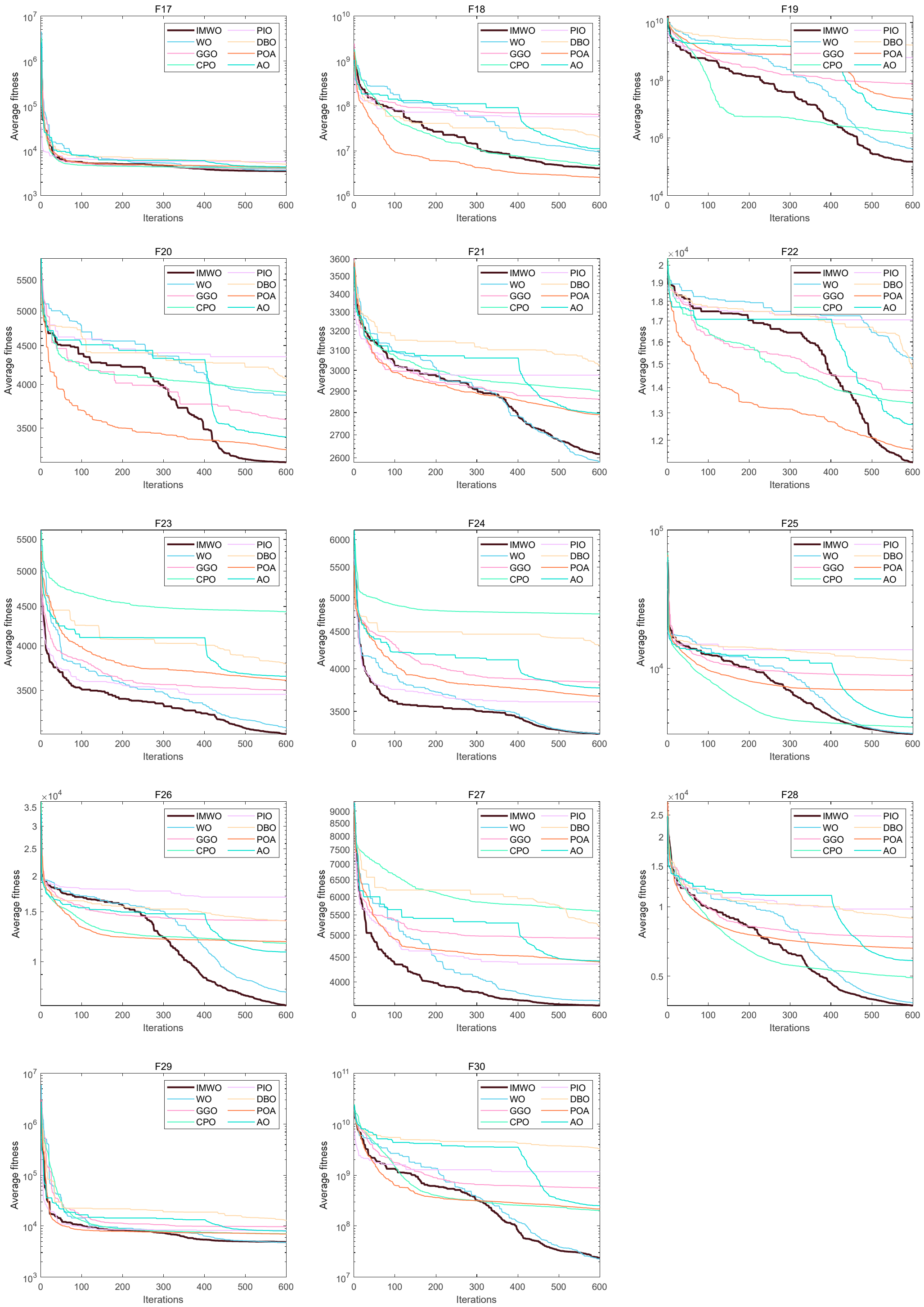


**Figure S3.** Figures of convergence curve comparison of various algorithms on CEC2017 functions (50-dimensional).

Figure S4 presents the performance evaluation of multiple algorithms on 50-dimensional CEC2017 functions. In the radar chart (Figure S4(a)), the black contour line of IMWO is closest to the center in most function directions, clearly outperforming other algorithms in most test scenarios. Figure S4(b) combines a

bar chart and a line chart to show the average ranking of each algorithm. IMWO ranks first with an average ranking of 1.34, while the original WO algorithm has a ranking of 2.69—indicating a significant performance gap between IMWO and WO. Other comparison algorithms (e.g., GGO with 5.34, PIO with 6.90) have much higher rankings, lagging far behind IMWO. This fully verifies that IMWO's improvement strategy effectively enhances its optimization capability, allowing it to maintain advantages even when facing the higher complexity of 50-dimensional problems.

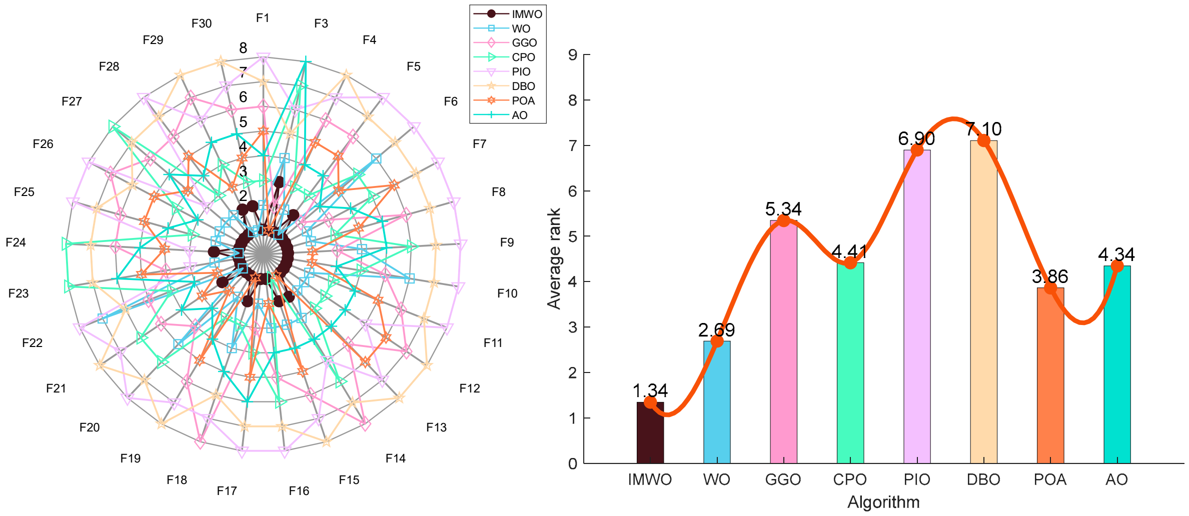


(a) (b)

**Figure S4.** (a) Figure of radar chart of various algorithms on CEC2017 functions, (b) Figure of ranking chart of various algorithms on CEC2017 functions (50-dimensional).

## 3. CEC2022 Test Functions（20-dimensional）

Figure S5 illustrates the average fitness variation of multiple algorithms across CEC2022 20-dimensional test functions (F1-F12) with iterations. Evidently, on most functions, IMWO outperforms WO and others: its average fitness drops faster in early iterations and stabilizes at a lower level later. This shows IMWO has better convergence speed and optimization accuracy than WO. While other algorithms perform differently across functions, IMWO's advantage is distinct—its improved strategy boosts optimization ability, letting it converge to optimal solutions quickly and compete well in complex 20D CEC2022 tasks.

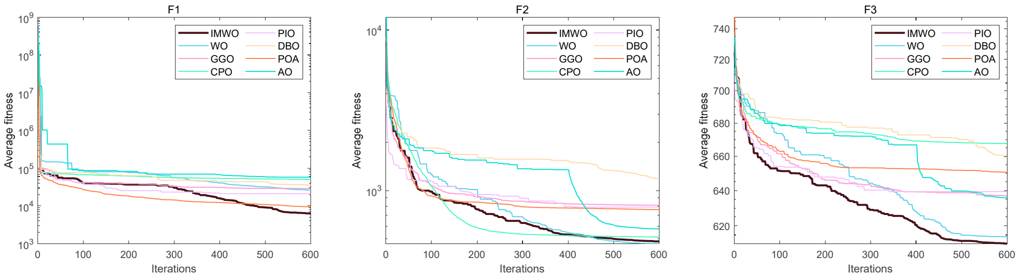

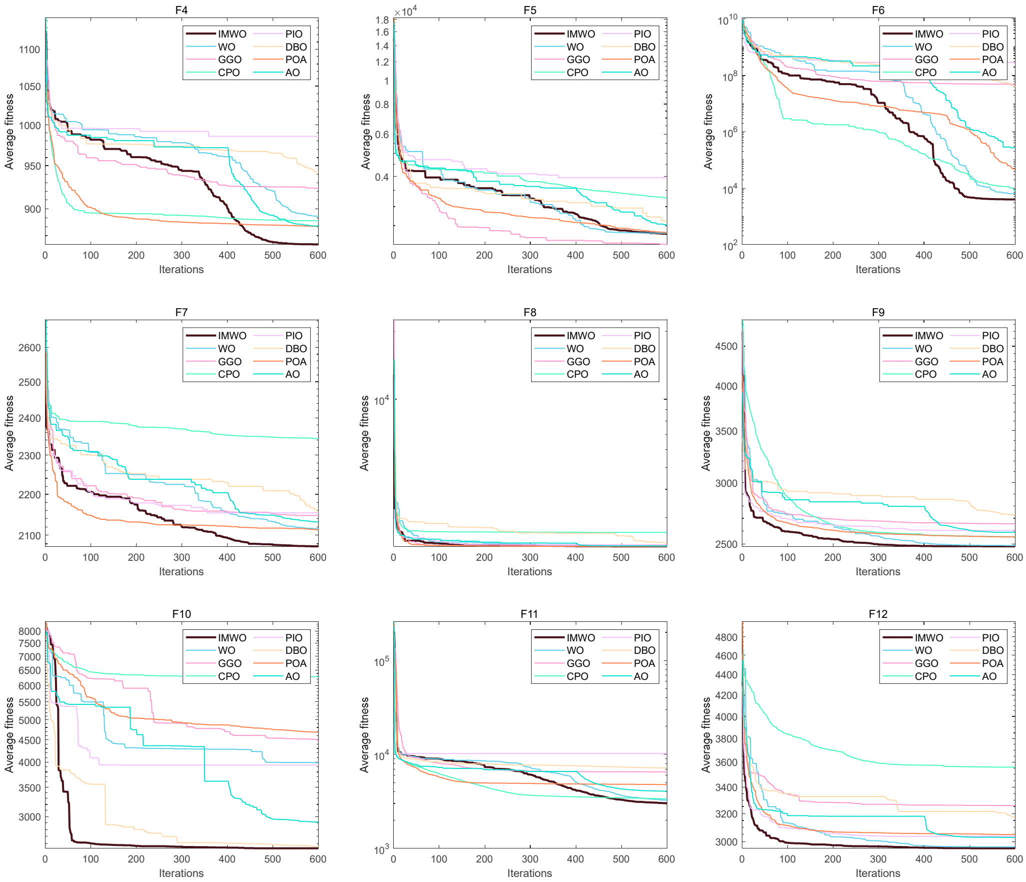


**Figure S5.** Figures of convergence curve comparison of various algorithms on CEC2022 functions(20-dimensional).

Figure S6 is the algorithm performance comparison chart. In the radar chart (a), following the logic that **closer to the center indicates better performance**, the black contour line of IMWO is closest to the center across most test function dimensions. In the ranking chart (b), based on the criterion that **lower values represent better comprehensive performance**, IMWO ranks first with an average ranking of 1.25, far outperforming WO (2.83) as well as other algorithms such as GGO and PIO. This directly demonstrates that IMWO leads the way in both single-function performance and overall performance.

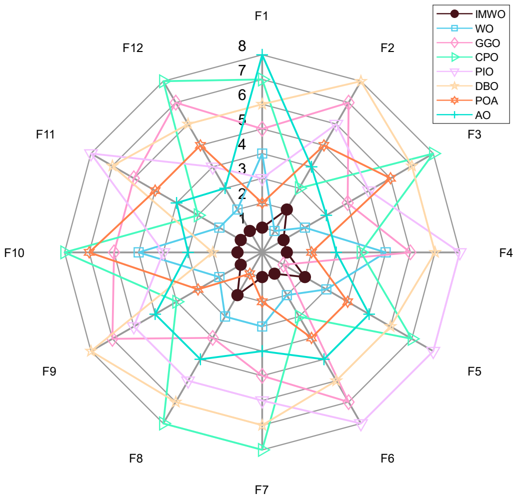


(a)

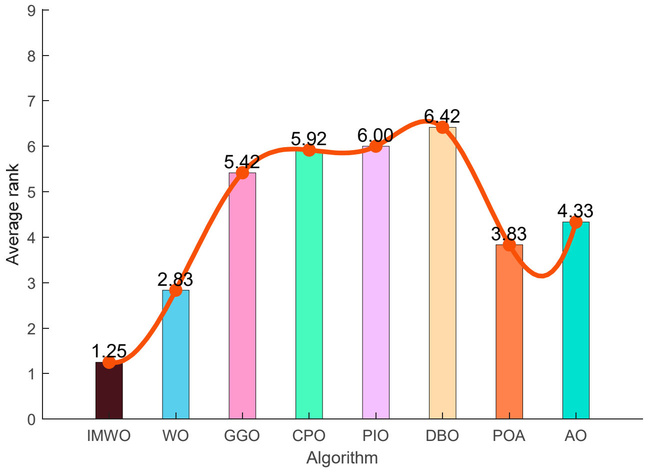


(b)

**Figure S6.** (a) Figure of radar chart of various algorithms on CEC2022 functions, (b) Figure of ranking chart of various algorithms on CEC2022 functions(20-dimensional).
